# The E3 ubiquitin ligase ARIH1 promotes antiviral immunity and autoimmunity by inducing mono-ISGylation and oligomerization of cGAS

**DOI:** 10.1038/s41467-022-33671-5

**Published:** 2022-10-10

**Authors:** Tian-Chen Xiong, Ming-Cong Wei, Fang-Xu Li, Miao Shi, Hu Gan, Zhen Tang, Hong-Peng Dong, Tianzi Liuyu, Pu Gao, Bo Zhong, Zhi-Dong Zhang, Dandan Lin

**Affiliations:** 1 Department of Gastrointestinal Surgery, Medical Research Institute, Frontier Science Center of Immunology and Metabolism, Zhongnan Hospital of Wuhan University, Wuhan University, Wuhan, China; 2 Chongqing International Institute for Immunology, Chongqing, China; 3 TaiKang Center for Life and Medical Sciences, Wuhan University, Wuhan, China; 4 Wuhan Research Center for Infectious Diseases and Cancer, Chinese Academy of Medical Sciences, Wuhan, China; 5 CAS Key Laboratory of Infection and Immunity, Institute of Biophysics, Chinese Academy of Sciences, Beijing, China; 6 Department of Virology, College of Life Sciences, Wuhan University, Wuhan, China; 7 Cancer Center, Renmin Hospital of Wuhan University, Wuhan, China

**Keywords:** Pattern recognition receptors, Autoimmunity, Inflammation, Innate immunity

## Abstract

The cytosolic DNA sensor cyclic GMP-AMP synthase (cGAS) plays a critical role in antiviral immunity and autoimmunity. The activity and stability of cGAS are fine-tuned by post-translational modifications. Here, we show that ariadne RBR E3 ubiquitin protein ligase 1 (ARIH1) catalyzes the mono-ISGylation and induces the oligomerization of cGAS, thereby promoting antiviral immunity and autoimmunity. Knockdown or knockout of ARIH1 significantly inhibits herpes simplex virus 1 (HSV-1)- or cytoplasmic DNA-induced expression of type I interferons (IFNs) and proinflammatory cytokines. Consistently, tamoxifen-treated ER-Cre;*Arih1*^fl/fl^ mice and *Lyz2*-Cre; *Arih1*^fl/fl^ mice are hypersensitive to HSV-1 infection compared with the controls. In addition, deletion of ARIH1 in myeloid cells alleviates the autoimmune phenotypes and completely rescues the autoimmune lethality caused by TREX1 deficiency. Mechanistically, HSV-1- or cytosolic DNA-induced oligomerization and activation of cGAS are potentiated by ISGylation at its K187 residue, which is catalyzed by ARIH1. Our findings thus reveal an important role of ARIH1 in innate antiviral and autoimmune responses and provide insight into the post-translational regulation of cGAS.

## Abstract (web summary)

The activity and stability of the cytosolic DNA sensor cGAS, a key mediator of innate antiviral immunity and autoimmunity, is fine-tuned by post-translational modifications. Here, the authors demonstrate that the ubiquitin E3 ligase ARIH1 catalyzes the mono-ISGylation of cGAS and promotes its oligomerization in response to viral and self DNA.

## Introduction

The innate immune system detects viral nucleic acids through pattern-recognition receptors (PRRs) including RIG-I-like receptors, Toll-like receptors (TLRs), and cytosolic DNA sensors to initiate antiviral immune responses^1,2^. To date, multiple cytoplasmic DNA sensors have been identified, among which the cyclic guanosine monophosphate-adenosine monophosphate (cGAMP) synthase (cGAS) is universally required for the recognition of viral DNA in distinct types of cells and thus serves as a major DNA sensor in the cytoplasm^3,4^. Upon binding to DNA, cGAS is activated through conformational transitions, resulting in the formation of a catalytically competent and an accessible nucleotide-binding pocket for ATP and GTP^5–8^. Structural and biochemical analyses have revealed that the binding of cGAS to DNA promotes cGAS oligomerization—a hallmark of cGAS activation—and efficiently catalyzes the conversion of ATP and GTP into cGAMP^6–10^. Subsequently, cGAMP binds to and activates the adaptor protein mediator of IRF3 activation (MITA, also known as STING and ERIS), which induces the expression of an array of downstream genes including type I interferons (IFNs) and proinflammatory cytokines to elicit cellular antiviral immune responses^11–13^. Consistently, cGAS deficiency abolishes the production of cGAMP and type I IFNs after HSV-1 infection and cytoplasmic DNA challenge, and mice deficient in cGAS are hypersensitive to HSV-1 infection^3^. In addition to viral DNAs, cGAS also recognizes and is activated by self DNA derived from mitochondrial DNA (mtDNA) and endogenous retroelements-derived DNA^14,15^. Such self-DNA molecules are normally degraded by multiple cytoplasmic DNase, including three prime repair exonuclease 1 (TREX1) and DNase II^16,17^. Loss-of-function mutations of TREX1 and DNase II have been reported to be highly connected with autoimmune diseases such as Aicardi-Goutieres syndrome, systemic lupus erythematosus, and retinal vasculopathy with cerebral leukodystrophy in humans^18,19^. Similarly, mice deficient in DNase II or TREX1 or mice carrying the loss-of-function TREX1 (*Trex1*^D18N/D18N^) produce overwhelming type I IFNs and ISGs and exhibit systemic lethal autoimmune phenotypes^16,17,20^. Notably, these autoimmune phenotypes are fully eliminated by depletion of cGAS, suggesting an indispensable role of cGAS in autoimmune diseases triggered by self-DNA^18^. Therefore, elucidating the regulatory mechanisms for cGAS activation would provide insight into the understanding of antiviral immunity and autoimmunity.

Post-translational modifications such as ubiquitination and ubiquitin-like modifications have been reported to tightly control the activity and stability of cGAS and finely tune cGAS-mediated immune responses^2^. TTLL4 and TTLL6 catalyze glutamylation of cGAS to impede the DNA binding activity and synthase activity of cGAS, which is relieved by CCP5- and CCP6-mediated deglutamylation^21^. Mono-ubiquitination of cGAS mediated by E3 ubiquitin ligases RINCK and TRIM56 and K27-linked polyubiquitination of cGAS catalyzed by RNF185 have been shown to promote the synthesis of cGAMP and the expression of downstream genes after HSV-1 infection^22–24^. In addition, an unknown E3 ubiquitin ligase constitutively catalyzes K48-linked ubiquitination of cGAS and promotes its degradation through the proteasome-dependent pathway in the resting cells and after HSV-1 infection, which is counteracted by the deubiquitinating enzyme USP29^25^. Consequently, knockout of USP29 results in downregulation of cGAS in cells and organs that impairs antiviral immune responses and rescues autoimmune lethality caused by TREX1 deficiency. A recent study has shown that the E3 ubiquitin ligase TRIM38 targets cGAS for SUMOylation, which suppresses the K48-linked polyubiquitination of cGAS in a manner of physical hindrance and stabilizes cGAS in resting cells and early phase of HSV-1-infected cells^26^. More recently, it has been reported that RNF111 mediates NEDDylation of cGAS, which leads to the dimerization and subsequent activation of cGAS^27^. Such SUMOylation and NEDDylation of cGAS indicate that ubiquitin-like modifications regulate the activity and stability of cGAS^28,29^.

ISG15 is an IFN-inducible protein composed of two ubiquitin-like domains linked by a short hinge region and unconjugated ISG15 can regulate viral replication and host antiviral responses through non-covalent protein-protein interactions and its action as a cytokine^30^. In addition, the mature ISG15 protein bares a C-terminal LRLRGG motif that can be covalently conjugated to the lysine residues of substrates by a three-step process known as ISGylation involving ubiquitin-activating enzyme E1 homolog (UBE1L, also known as UBA7), E2 (UBCH8, also known as UBE2L6), and E3 ligases. Mice lacking UBE1L exhibit hypersensitivity to viral infections^31,32^. *Isg15*^−/−^ mice are more susceptible to viral infections, which can be rescued by expressing wild-type ISG15, but not a mutant form of ISG15 that cannot form conjugates, from the virus genome^33^. Similar to ubiquitination, ISGylation is a reversible process and counteracted by USP18^34^. Consistently, USP18^C61A/C61A^ knock-in mice (in which the deconjugating activity of USP18 is disrupted) exhibit elevated ISGylation and increased viral resistance, which is completely reversed by additional deletion of ISG15^35^, indicating that ISGylation of substrates inhibits viral infections and promotes antiviral immunity. Proteomic studies have identified hundreds of host proteins that are ISGylated upon interferon stimulation^36^. However, only a subset of proteins has been validated for ISGylation that has been investigated in the context of viral infections^37–39^. For example, ISGylation of IRF3, STAT1, and PKR promote their stability or activity to increase the production of type I IFNs and ISGs^38–41^, whereas ISG15 modification of RIG-I promotes its degradation to turn down cellular antiviral responses^37^. Whether and how cGAS undergoes ISGylation to regulate antiviral immunity and autoimmunity are completely unknown.

Ariadne RBR E3 ubiquitin protein ligase 1 (ARIH1, also known as HHARI) is a RING-between-RING (RBR)-type ubiquitin E3 ligase that associates with two E2 ubiquitin-conjugating enzymes UBCH7 and UBCH8^42^. Early studies have shown that ARIH1 induces ubiquitination and ISGylation of the translation initiation factor 4E homologous protein (4EHP) in the presence of UBCH7 and UBCH8, respectively^43,44^, suggesting that ARIH1 possesses dual catalytic activities in the presence of different E2 enzymes. Interestingly, recent structural analyses suggest that ARIH1 is a component of Cullin-RING ligases (CRLs) and forms a unique E3-E3 platform with Cullin-associated RBX1, and thereby preferentially catalyzes mono-ubiquitination of diverse substrates presented on various substrate receptors (such as F-box proteins and BTB proteins)^45–47^. Specifically, the NEDD8-Cullin-RBX1 complex activates auto-inhibited ARIH1 and the UBCH7-conjugated ubiquitin is firstly transferred to ARIH1 and then to the lysine residues of substrates^47^. Subsequently, the substrates either leave the CRL complex or undergo further ubiquitination on the mono-ubiquitinated lysine residues by CRLs^46,47^. However, whether and how ARIH1 mediates ISGylation of substrates through CRLs remain to be investigated.

In this study, we identified ARIH1 as a cGAS-interacting E3 ubiquitin ligase that mediates mono-ISGylation of cGAS and promotes its oligomerization after HSV-1 infection. Knockout or knockdown of ARIH1 attenuates cellular antiviral responses against HSV-1 and knockout of ARIH1 leads to hypersensitivity to HSV-1 infection and alleviates the autoimmune phenotypes in *Trex1*^−/−^ mice. Mechanistically, ARIH1 directly catalyzes mono-ISGylation at K187 of cGAS in vitro and in cells after HSV-1 infection, which relieves K187-mediated inhibitory effects on the oligomerization of cGAS. These findings highlight a regulatory mechanism of cGAS activation mediated by ARIH1 and provide potential targets for viral infection-caused diseases and autoimmune disorders.

## Results

### Identification of ARIH1 as a cGAS-interacting E3 ubiquitin ligase

**Fig. 1:**
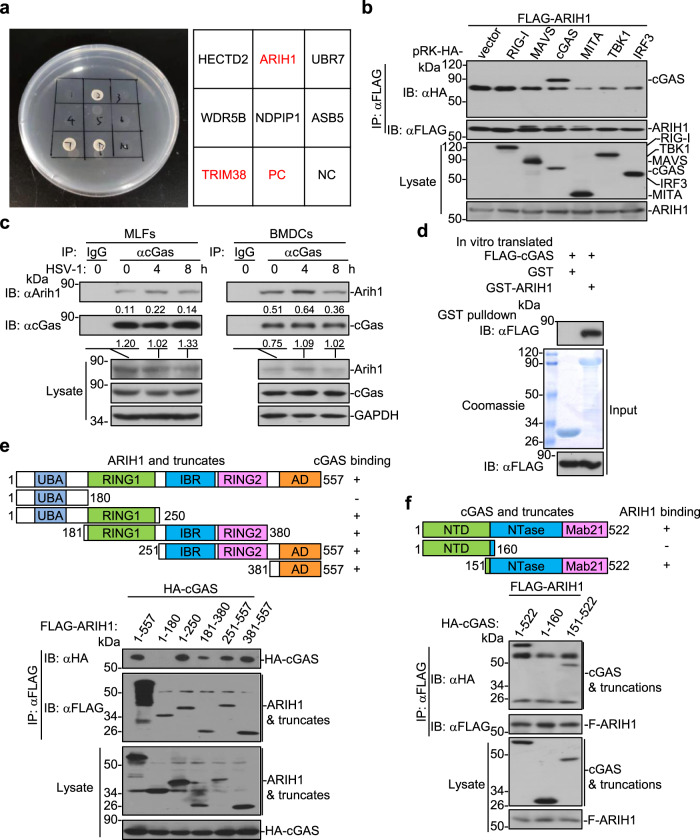
ARIH1 interacts with cGAS. **a** Yeast-two hybrid assays with AH109 cells that were co-transformed with pGBT9-cGAS and pGADT7-E3 constructs. PC positive control (pGBT9-MAVS and pGAD7-RNF115); NC negative control (pGBT9-cGAS and pGADT7 empty vector). **b** Immunoprecipitation (with anti-FLAG) and immunoblot analysis (with anti-FLAG or anti-HA) in HEK293 cells that were transfected with plasmids encoding FLAG-ARIH1 and HA-tagged, RIG-I, MAVS, cGAS, MITA, TBK1 or IRF3 for 24 h. **c** Immunoprecipitation (with control IgG or anti-cGAS) and immunoblot analysis (with anti-cGAS, anti-ARIH1, or anti-GAPDH) in MLFs (left) or BMDCs (right) that were left uninfected or infected with HSV-1 for 4–8 h. **d** Immunoblot analysis (with anti-FLAG) of the GST-pulldown precipitants from the incubation of GST or GST-ARIH1 (5 μg) and in vitro translated FLAG-cGAS. The input GST and GST-ARIH1 were subject to SDS-PAGE followed by Coomassie brilliant blue staining. **e**, **f** Immunoprecipitation (with anti-FLAG) and immunoblot analysis (with anti-FLAG or anti-HA) in HEK293 cells that were transfected with plasmids encoding HA-cGAS and FLAG-tagged ARIH1 or truncates (**e**) or with plasmids encoding FLAG-ARIH1 and HA-tagged cGAS or mutants (**f**) for 24 h. UBA Ubiquitin-associated domain, RING1 RING domain 1, IBR in-between RING domain, RING2 RING domain 2, AD Ariadne domain, NTD N-terminal domain, NTase nucleotidyl transferase, Mab21 Mab-21 domain. Data are representative of three independent experiments (**a**–**f**). Source data are provided as a Source Data file.

cGAS is a cytosolic DNA sensor that critically mediates antiviral immunity and autoimmunity^3,4^. The activity and stability of cGAS are strictly controlled by various posttranslational modifications^2^. To identify cGAS-interacting E3 ligases that potentially regulate the modifications and functions of cGAS, we performed yeast-two hybrid screening with pGBT9-cGAS and ~200 pGADT7-E3 constructs in AH109 cells^48^. This effort led to the identification of ARIH1 (also known as HHARI) as a cGAS-interacting E3 in yeast cells (Fig. 1a). Results from transient transfection and co-immunoprecipitation assays suggested that ARIH1 selectively interacted with cGAS but not with other proteins including RIG-I, MAVS, MITA, TBK1, or IRF3 in HEK293 cells (Fig. 1b). We next examined the endogenous association between cGAS and ARIH1 and found that cGAS interacted with ARIH1 constitutively in primary murine lung fibroblasts (MLFs) and bone marrow-derived dendritic cells (BMDCs) (Fig. 1c). The association between cGAS and ARIH1 was substantiated at early time points and then impaired at late time points after HSV-1 infection (Fig. 1c). In addition, results from Glutathione S-transferase (GST) pulldown assays revealed a direct interaction between the GST-tagged ARIH1 purified from bacteria and FLAG-cGAS obtained from an in vitro transcription and translation kit (Fig. 1d). Domain mapping analysis showed that multiple domains except for the N-terminal UBA domain of ARIH1 interacted with cGAS, while the C-terminal domain of cGAS (aa151-552) interacted with ARIH1 in HEK293 cells (Fig. 1e, f). Together, these data suggest that ARIH1 interacts with cGAS directly and constitutively under homeostatic conditions and after HSV-1 infection.

### Knockdown of ARIH1 inhibits HSV-1-triggered expression of downstream genes

ARIH1 is a component of the CRL system and regulates cancer progression and xenophagy of cytosolic bacteria^46,49–51^. Since ARIH1 interacts with cGAS that mediates innate antiviral responses, we investigated the role of ARIH1 in HSV-1- or cytoplasmic DNA-triggered immune signaling. We designed two shRNAs targeting ARIH1, both of which downregulated the protein levels of endogenous ARIH1 in HEK293 cells (Supplementary Fig. 1a). In addition, both of the shRNAs inhibited cGAS plus MITA- but not MITA-mediated activation of the ISRE enhancer in luciferase reporter assays in HEK293 cells (Supplementary Fig. 1b). We next chose shARIH1#1 for subsequent experiments and found that knockdown of ARIH1 significantly impaired HSV-1-induced expression of *IFNB*, *ISG56*, *CCL5* and *IP10* in THP-1 cells (Supplementary Fig. 1c). In addition, knockdown of ARIH1 substantially inhibited the expression of *IFNB*, *ISG15*, *CCL5*, and *IP10* after transfection of various DNA, but barely affected cGAMP-induced expression of *IFNB*, *IL6*, *CCL5*, *ISG15*, and *IP10* (Supplementary Fig. 1d, e). Consistent with these observations, HSV-1- but not cytoplasmic cGAMP-induced phosphorylation of IRF3, TBK1, and p65 was impaired by knockdown of ARIH1 in THP-1 cells (Supplementary Fig. 1f). These data together suggest that ARIH1 positively regulates DNA virus- or cytoplasmic DNA-triggered innate immune signaling at the level of cGAS.

### Knockout of ARIH1 inhibits cellular antiviral responses against HSV-1

The *C. elegans* ARI1s (homolog of human ARIH1) has been implicated in fertility and oogenesis during development^52^. Germline knockout of ARIH1 in mice might affect the development and growth of mice. To further investigate the role of ARIH1 in antiviral immune responses in vivo, we generated *Arih1*^fl/+^ mice by CRISPR/Cas9- mediated genome editing (Supplementary Fig. 2a). Results from Southern blot analysis indicated that the targeting vector was successfully recombined with the wild-type allele (Supplementary Fig. 2b). Cre recombinase-mediated deletion of the exon 3 between flanking loxp sites would lead to early translational termination of ARIH1 to generate a 165 amino-acid peptide or result in *Arih1* mRNA instability (Supplementary Fig. 2c, d). The *Arih1*^fl/+^ mice were then crossed with *Lyz2*-Cre mice to obtain the *Lyz2*-Cre;*Arih1*^fl/fl^ mice. The differentiation of *Lyz2*-Cre;*Arih1*^fl/fl^ bone marrow cells into BMDCs and BMDMs was similar to the *Arih1*^fl/fl^ counterparts in the presence of GM-CSF and M-CSF cultures, respectively (Supplementary Fig. 2e). The number and composition of lymphocytes in multiple organs including thymus, inguinal lymph nodes, and spleen were comparable between the *Lyz2*-Cre *Arih1*^fl/fl^ mice and the *Arih1*^fl/fl^ mice (Supplementary Fig. 2f–h). In addition, the percentages and numbers of myeloid CD11b^+^ and CD11c^+^ populations in spleen from *Lyz2*-Cre;*Arih1*^fl/fl^ mice were similar to those from *Arih1*^fl/fl^ mice (Supplementary Fig. 2h). Together, these results suggest that ARIH1 in myeloid cells is dispensable for the in vitro differentiation of BMDCs and BMDMs and the in vivo development and homeostasis of various immune cells.

**Fig. 2:**
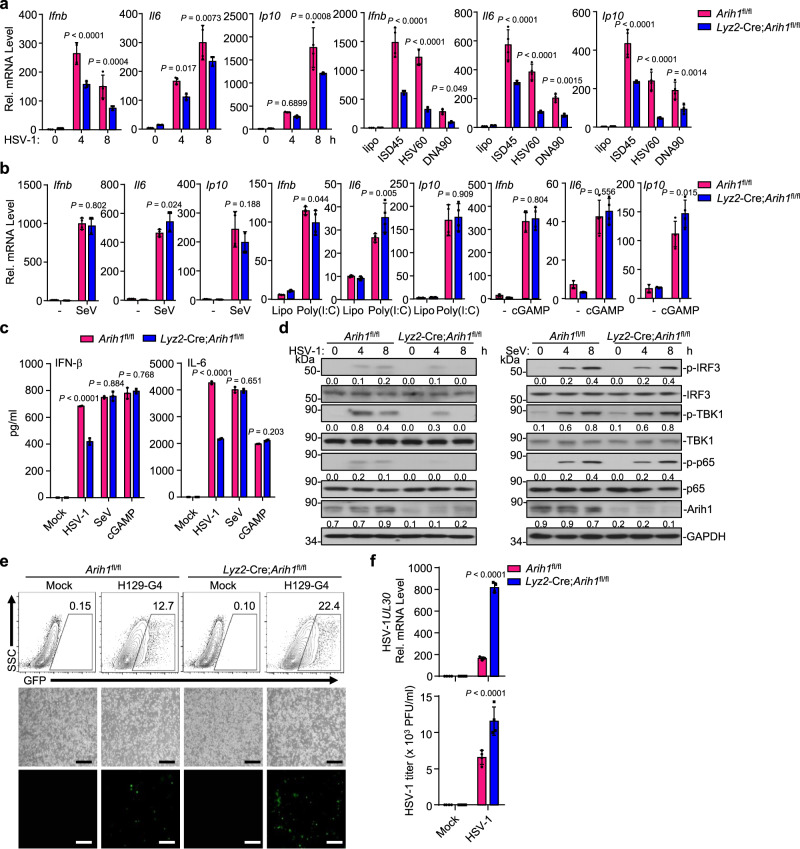
Knockout of ARIH1 inhibits cellular antiviral responses against HSV-1. **a** qRT-PCR analysis of *Ifnb, Il6* and *Ip10* mRNA in *Arih1*^fl/fl^ and *Lyz2*-Cre;*Arih1*^fl/fl^ BMDCs infected with HSV-1 for 0–8 h or transfected with ISD45, HSV60 or DNA90 (1 μg) for 8 h. **b** qRT-PCR analysis of *Ifnb, Il6* and *Ip10* mRNA in *Arih1*^fl/fl^ and *Lyz2*-Cre;*Arih1*^fl/fl^ BMDCs infected with SeV for 0–8 h or transfected with poly(I:C) (1 μg) or cGAMP (1 μg) for 0–4 h. **c** ELISA analysis of IFN-β and IL-6 in the supernatants of *Arih1*^fl/fl^ and *Lyz2*-Cre;*Arih1*^fl/fl^ BMDCs infected with HSV-1 or SeV for 12 h, or transfected with cGAMP (1 μg) for 12 h. **d** Immunoblot analysis of total and phosphorylated (p-) IRF3, TBK1 and p65, and ARIH1 and GAPDH in *Arih1*^fl/fl^ and *Lyz2*-Cre;*Arih1*^fl/fl^ BMDCs infected with HSV-1 (left) or SeV (right) for 0–8 h. **e** Flow cytometric analysis (upper flow charts) and fluorescent microscopy imaging (lower images) of the replication of H129-G4 (MOI = 0.5) in *Arih1*^fl/fl^ and *Lyz2*-Cre;*Arih1*^fl/fl^ BMDCs that were left uninfected or infected with H129-G4 for 1 h followed by twice PBS wash and culture in full medium for 24 h. Numbers adjacent to the outlined areas indicate percentages of GFP^+^ BMDCs. Scale bars represent 200 μm. **f** qRT-PCR analysis of HSV-1 *UL30* mRNA in *Arih1*^fl/fl^ and *Lyz2*-Cre;*Arih1*^fl/fl^ BMDCs (upper graph) and plaque assays analyzing HSV-1 titers in the supernatants of *Arih1*^fl/fl^ and *Lyz2*-Cre;*Arih1*^fl/fl^ BMDCs (lower graph) infected with HSV-1 (MOI = 0.5) for 1 h followed by twice PBS wash and culture in full medium for 24 h. Data are representative of three (a-b) or two independent experiments (**c**–**f**). Graphs show mean ± S.D. (*n* = 4 for (**a**, **b**, **f**) or *n* = 3 for (**c**), biologically independent experiments). Statistical significance was determined using two-tailed Student’s *t* tests in (**a**–**c**, **f**). The quantitative anaylsis is derive from the same experiment and that blots were processed in parallel in (**d**). Source data are provided as a Source Data file.

We next examined the effect of ARIH1 deficiency on HSV-1- or cytoplasmic DNA-triggered signaling in primary murine BMDCs. We found that knockout of ARIH1 significantly impaired HSV-1- or cytoplasmic DNA- but not SeV-, poly(I:C)-, or cGAMP-induced expression of *Ifnb*, *Il6*, and *Ip10* and production of IFN-β and IL-6 in BMDCs (Fig. 2a–c). Consistently, HSV-1- but not SeV-induced phosphorylation of TBK1, IRF3, and p65 was substantially inhibited in the *Lyz2*-Cre;*Arih1*^fl/fl^ BMDCs compared to the *Arih1*^fl/fl^ BMDCs (Fig. 2d), suggesting that ARIH1 selectively promotes HSV-1- and cytoplasmic DNA-triggered cGAS-dependent innate immune signaling. The virus-triggered expression of downstream genes plays a critical role in restricting the replication of viruses. Consistent with this notion, the replication of H129-G4 (a GFP-tagged HSV-1)^48^ and HSV-1 was significantly potentiated in *Lyz2*-Cre;*Arih1*^fl/fl^ BMDCs compared to *Arih1*^fl/fl^ BMDCs as revealed by the GFP signals determined by flow cytometry and fluorescent microscopy analyses, the expression levels of HSV-1 *UL30* gene determined by qRT-PCR assays, and the HSV-1 titers in the supernatants determined by plaque assays (Fig. 2e, f). These data suggested that ARIH1 is required for optimal antiviral immune responses to restrict HSV-1 replication in myeloid cells.

We also obtained ROSA26-CreERT2 (here referred to as Cre-ER) *Arih1*^fl/fl^ mice by crossing the *Arih1*^fl/fl^ mice and Cre-ER mice. Intraperitoneal injection of tamoxifen led to efficient deletion of ARIH1 in various organs including brain, lung, heart, and liver (Supplementary Fig. 3a), suggesting that the strategy to knockout Arih1 in mice is reliable and successful. Then we prepared the primary murine lung fibroblasts (MLFs) from Cre-ER;*Arih1*^fl/fl^ and Cre-ER mice and treated these cells with 4-hydroxytamoxifen (4-OHT) followed by HSV-1 infection or transfection of various ligands. Results from qRT-PCR and ELISA assays revealed that HSV-1- or cytoplasmic DNA- but not SeV-, cytoplasmic poly(I:C)-, or cGAMP-induced expression of *Ifnb*, *Ifna4* or *Ccl5* and production of IFN-β and IL-6 were significantly lower in Cre-ER;*Arih1*^fl/fl^ MLFs than in Cre-ER MLFs (Supplementary Fig. 3b, d). Consistently, HSV-1- but not SeV-induced phosphorylation of TBK1, IRF3, and p65 was markedly decreased in the Cre-ER;*Arih1*^fl/fl^ MLFs compared to the Cre-ER MLFs (Supplementary Fig. 3e). As expected, the replication of H129-G4 and HSV-1 was potentiated in Cre-ER;*Arih1*^fl/fl^ MLFs compared to Cre-ER MLFs (Supplementary Fig. 3f, g). Collectively, these data demonstrate that ARIH1 promotes cellular antiviral responses against HSV-1 in various types of primary murine cells.

### ARIH1 deficiency leads to increased susceptibility to lethal HSV-1 infection

**Fig. 3:**
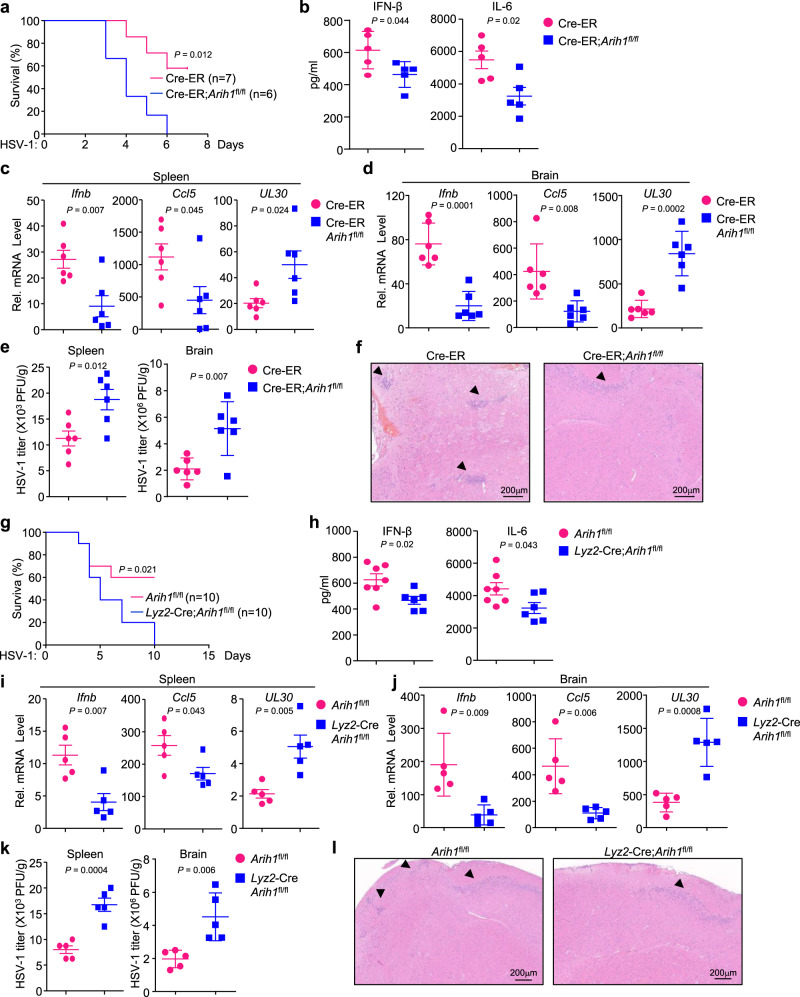
ARIH1 deficiency leads to increased susceptibility to HSV-1 infection. **a** Survival (Kaplan–Meier curve) of Cre-ER (*n* = 7) and Cre-ER;*Arih1*^fl/fl^ mice (*n* = 6) intraperitoneally injected with tamoxifen for five successive days and rested for 5 days followed by intraperitoneal injection with HSV-1. **b** ELISA analysis of IFN-β and IL-6 in the sera of Cre-ER (*n* = 5) and Cre-ER;*Arih1*^fl/fl^ mice (*n* = 5) treated as in (**a**) and intraperitoneally injected with HSV-1 for 12 h. **c**, **d** qRT-PCR analysis of *Ifnb*, *Ccl5*, and *UL30* mRNA in the spleen (**c**) or brain (**d**) from Cre-ER (*n* = 6) and Cre-ER;*Arih1*^fl/fl^ mice (*n* = 6) treated as in (**a**) and intraperitoneally injected with HSV-1 for 3 days. **e** Plaque assays analyzing HSV-1 titers in the spleen or brain from Cre-ER (*n* = 6) and Cre-ER;*Arih1*^fl/fl^ mice (*n* = 6) treated as in (**c**). **f** Hematoxylin-eosin (HE) staining of brain sections of Cre-ER (*n* = 4) and Cre-ER;*Arih1*^fl/fl^ mice (*n* = 4) treated as in (**a**) followed by intracerebral injection of HSV-1 for 3 days. **g** Survival of *Arih1*^fl/fl^ (*n* = 10) and *Lyz2*-Cre;*Arih1*^fl/fl^ mice (*n* = 10) intraperitoneally injected with HSV-1. **h** ELISA analysis of IFN-β and IL-6 in the sera of *Arih1*^fl/fl^ (*n* = 7) and *Lyz2*-Cre;*Arih1*^fl/fl^ mice (*n* = 6) intraperitoneally injected with HSV-1 for 12 h. **i**, **j** qRT-PCR analysis of *Ifnb*, *Ccl5*, and *UL30* mRNA in the spleen and brain from *Arih1*^fl/fl^ (*n* = 5) and *Lyz2*-Cre;*Arih1*^fl/fl^ mice (*n* = 5) intraperitoneally injected with HSV-1 for 3 days. **k** Plaque assays analyzing HSV-1 titers in the spleen or brain from *Arih1*^fl/fl^ (*n* = 5) and *Lyz2*-Cre *Arih1*^fl/fl^ mice (*n* = 5) intraperitoneally injected with HSV-1 for 3 days. **l** HE staining of brain sections of *Arih1*^fl/fl^ (*n* = 4) and *Lyz2*-Cre *Arih1*^fl/fl^ mice (*n* = 4) intracerebrally injected with HSV-1 respectively for 3 days. 1 × 10^7^ PFU and 1 × 10^6^ PFU HSV-1 were injected with per mice in (**a**–**e**, **g**–**k**, **f**). **l** respectively. Data are of combined two independent experiments (**a**, **g**) or representative of two independent experiments (**b**–**f**, **h**–**l**). Graphs show mean ± S.D. (*n* = 5 for (**b**, **i**–**k**), or *n* = 6 for (**c**–**e**), or *n* = 7 and 6 for (**h**), biologically independent experiments). Statistical significance was determined using two-tailed Student’s *t* tests in (**b**–**e**, **h**–**k**). Source data are provided as a Source Data file.

We next examined the physiological role of ARIH1 in defense against HSV-1 infection in mice. The Cre-ER;*Arih1*^fl/fl^ mice and the Cre-ER mice were intraperitoneally injected with tamoxifen (80 mg/kg body weight) for five successive days, and 5 days later the mice were infected with sub-lethal HSV-1 (1 × 10^7^ PFU per mouse) via intraperitoneal (i.p.) route or HSV-1 (1 × 10^6^ PFU per mouse) via intracranial (i.c.) route. As shown in Fig. 3a, the Cre-ER;*Arih1*^fl/fl^ mice started to die at day 3 after HSV-1 infection and all the mice died at day 6 after HSV-1 infection. In contrast, the Cre-ER mice started to die at the fourth day after HSV-1 infection and 4 out of 7 mice survived at day 8 after HSV-1 infection (Fig. 3a). Moreover, the concentrations of IFN-β and IL-6 were significantly lower in the sera of Cre-ER;*Arih1*^fl/fl^ mice than in Cre-ER mice at 12 h after HSV-1 infection (Fig. 3b). The levels of *Ifnb* and *Ccl5* were decreased and the levels of HSV-1 *UL30* gene were increased in the spleen or brain of Cre-ER;*Arih1*^fl/fl^ mice compared to Cre-ER mice at day 3 after HSV-1 infection (Fig. 3c, d). In addition, the HSV-1 titers in the spleen or brain of Cre-ER;*Arih1*^fl/fl^ mice were higher than in the spleen or brain of Cre-ER mice at day 3 after HSV-1 infection (Fig. 3e). Hematoxylin-eosin staining analysis of brain tissues showed that there were fewer infiltrated cells in the brains of Cre-ER;*Arih1*^fl/fl^ mice than in the brains of control mice after HSV-1 infection (Fig. 3f). Similarly, deletion of ARIH1 by *Lyz2*-Cre led to increased susceptibility to sub-lethal HSV-1 infection (Fig. 3g), inhibited the production of IFN-β and IL-6 in the sera (Fig. 3h), and impaired the expression of *Ifnb* and *Ccl5* in the spleen or brain (Fig. 3i, j). Consistently, the levels of HSV-1 *UL30* gene and the HSV-1 titers in the spleen or brain of *Lyz2*-Cre;*Arih1*^fl/fl^ mice were significantly higher than those of *Arih1*^fl/fl^ mice (Fig. 3i–k). Finally, the infiltrated cells in the brains of *Lyz2*-Cre;*Arih1*^fl/fl^ mice were fewer than in the brains of their littermates after HSV-1 infection (Fig. 3l). Together, these data suggest that ARIH1 is required for optimal antiviral immune responses in mice.

### The enzyme activity of ARIH1 is required for antiviral signaling

Since the Cys357 of ARIH1 is the active site for ligase activity, we investigated whether re-introduction of ARIH1^C357S^ into ARIH1-deficient cells would restore the cellular antiviral responses. The Cre-ER;*Arih1*^fl/fl^ MLFs were transfected with an empty vector (Vec), ARIH1 or ARIH1^C357S^ followed by 4-OHT treatment and HSV-1 infection or transfection of various DNA. Results from qRT-PCR analyses suggested that reconstitution of wild-type ARIH1 but not ARIH1^C357S^ into Cre-ER *Arih1*^fl/fl^ MLFs restored- HSV-1- or cytoplasmic DNA-induced expression of *Ifnb*, *Il6* and *Isg56* (Supplementary Fig. 4a, b). In addition, the production of IFN-β and IL-6 in the supernatants and the phosphorylation of IRF3, TBK1, or p65 in cells were rescued by reconstitution of ARIH1 but not ARIH1^C357S^ (Supplementary Fig. 4c, d). Consistent with this notion, the replication of H129-G4 or HSV-1 was significantly inhibited in Cre-ER;*Arih1*^f/f^ MLFs reconstituted with ARIH1 but not with ARIH1^C357S^ as revealed by the GFP signals determined by flow cytometry and fluorescent microscopy imaging analyses, the expression levels of HSV-1 *UL30* gene determined by qPCR analysis, and the HSV-1 titers in the supernatants determined by plaque assays (Supplementary Fig. 4e, f). These data together suggest that ARIH1 promotes cellular antiviral responses dependently on its enzyme activity.

### ARIH1 promotes the oligomerization of cGAS

**Fig. 4:**
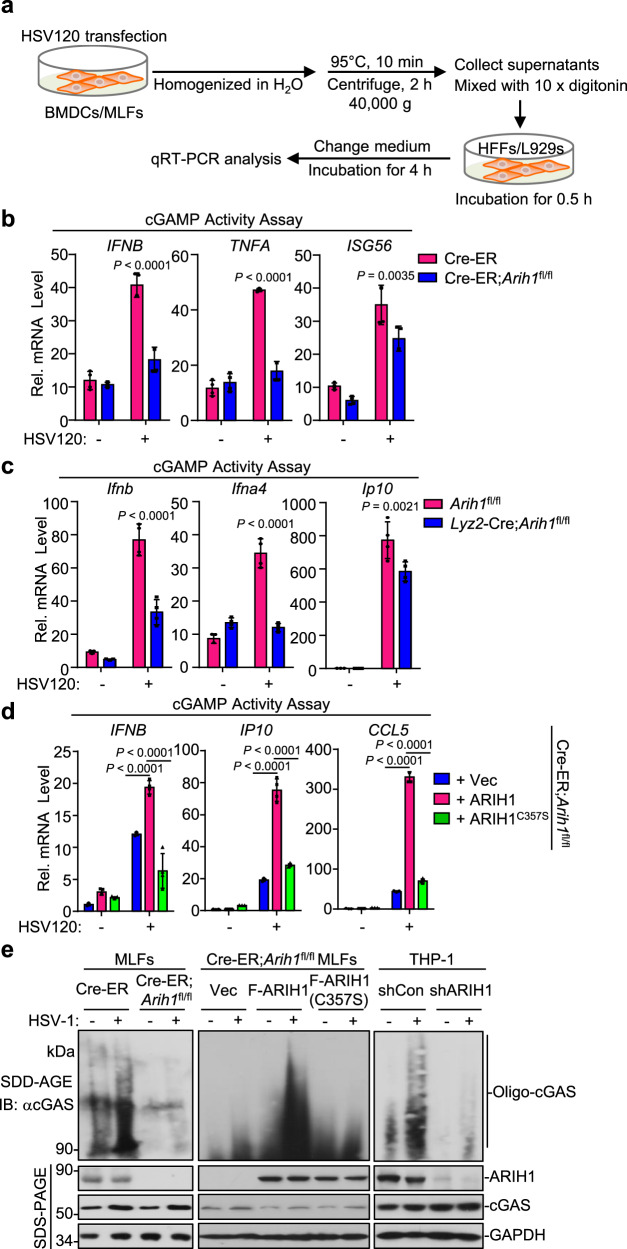
ARIH1 promotes the oligomerization and activation of cGAS. **a** A scheme showing cGAMP activity assay. The treated cells were harvested and homogenized in 1 mL sterile water followed by heating at 95 °C for 10 min. The homogenates were centrifuge at 40,000 × *g* for 2 h and the supernatants (900 μL) were mixed with 10 × digitonin (100 μL). The diluted supernatants were incubated with single-layered HFF cells or L929 cells (2 × 10^5^). Half an hour later, the supernatants were removed and full medium was added to culture HFFs or L929 cells for 4 h followed by qRT-PCR assays. **b** Cre-ER and Cre-ER;*Arih1*^fl/fl^ MLFs were treated with 4-hydroxytamoxifen (4OH Tam, 1 μM) for 3 days followed by transfection with HSV120 (1 μg) for 4 h. The cells were treated as in (**a**) and the supernatants were used to treat HFF cells for 6 h followed by qRT-PCR analysis. **c** *Arih1*^fl/fl^ and *Lyz2*-Cre;*Arih1*^fl/fl^ BMDCs were transfected with HSV120 (1 μg) for 4 h. The cell were treated as in (**a**) and the supernatants were used to treat L929 cells for 6 h followed by qRT-PCR analysis. **d** 4OHT-treated Cre-ER;*Arih1*^fl/fl^ MLFs treated were reconstituted with empty vector (Vec), ARIH1, or ARIH1(C357S) and transfected with HSV120 (1 μg) for 4 h. The cell cells were treated as in (**a**) and the supernatants were used to treat HFF cells for 6 h followed by qRT-PCR analysis. **e** Immunoblot assays with SDD-AGE to analyze the aggregation of cGAS and with SDS-PAGE to analyze the expression levels of the indicated proteins in 4OHT-treated Cre-ER and Cre-ER;*Arih1*^fl/fl^ MLFs (left panels), in 4OHT-treated Cre-ER;*Arih1*^fl/fl^ MLFs reconstituted with empty vector (Vec), ARIH1, or ARIH1^C357S^ (middle panels), or in THP-1 cells stably transfected with shCon or shARIH1 (right panels) followed by infection with HSV-1 for 4 h. Data are of representative of three independent experiments (**b**–**e**). Graphs show mean ± S.D. (*n* = 4 for (**b**–**d**), biologically independent experiments). Statistical significance was determined using two-tailed Student’s *t* tests in (**b**, **c**), or one-way ANOVA in d. Source data are provided as a Source Data file.

Because knockout of ARIH1 inhibited cytoplasmic DNA- but not the cGAMP-induced expression of type I IFNs and inflammatory genes in BMDCs or MLFs (Fig. 2b, c and Supplementary Fig. 3b, c), we reasoned that ARIH1 promoted cellular antiviral immune responses at the level of cGAS. To test this hypothesis, we performed the cGAMP activity assay by stimulating human foreskin fibroblasts (HFFs) or NCTC clone 929 clone of strain L (L929) mouse fibroblasts with the heat-resistant homogenates of ARIH1-sufficient or deficient cells that were transfected with DNA (Fig. 4a). Results from qRT-PCR analysis suggested that the homogenates from Cre-ER;*Arih1*^fl/fl^ MLFs that were transfected with DNA120 failed to induce expression of *IFNB*, *TNFA* and *ISG56* in HFF cells compared to those from Cre-ER MLFs (Fig. 4b). Similarly, the homogenates from DNA120-transfected *Arih1*^fl/fl^ BMDCs induced significantly higher expression of *Ifnb*, *Ifna4* and *Ip10* in L929 cells than those from DNA120-transfected *Lyz2*-Cre;*Arih1*^fl/fl^ BMDCs (Fig. 4c), indicating that knockout of ARIH1 compromises the cGAMP production in cells after transfection of DNA. In addition, the cGAMP activity was restored by reconstitution of ARIH1 but not ARIH1^C357S^ into 4-OHT-treated Cre-ER;*Arih1*^fl/fl^ MLFs (Fig. 4d). These data are consistent with the observations that reconstitution of ARIH1 but not ARIH1^C357S^ into ARIH1-deficient cells restored cytoplasmic DNA-induced expression of downstream genes (Supplementary Fig. 4a–c). Collectively, these data indicate that ARIH1 facilitates cGAS-mediated cGAMP production after cytoplasmic DNA stimulation.

Previous studies have demonstrated that HSV-1 infection or cytoplasmic DNA stimulation induces oligomerization of cGAS which represents a critical step for the activation of cGAS and the production of cGAMP^7^. Interestingly, we found that the HSV-1-induced oligomerization of cGAS was substantially impaired by knockdown of ARIH1 in THP-1 cells or by knockout of ARIH1 in MLFs (Fig. 4e). In addition, reconstitution of ARIH1 but not ARIH1^C357S^ into 4-OHT-treated Cre-ER;*Arih1*^fl/fl^ MLFs restored the oligomerization of cGAS after HSV-1 infection (Fig. 4e). Taken together, these data suggest that ARIH1 facilitates the oligomerization of cGAS and cGAS-mediated cGAMP production in cells after cytoplasmic DNA challenge or HSV-1 infection.

### Knockout of ARIH1 in myeloid cells rescues autoimmune lethality of *Trex1*^−/−^ mice

**Fig. 5:**
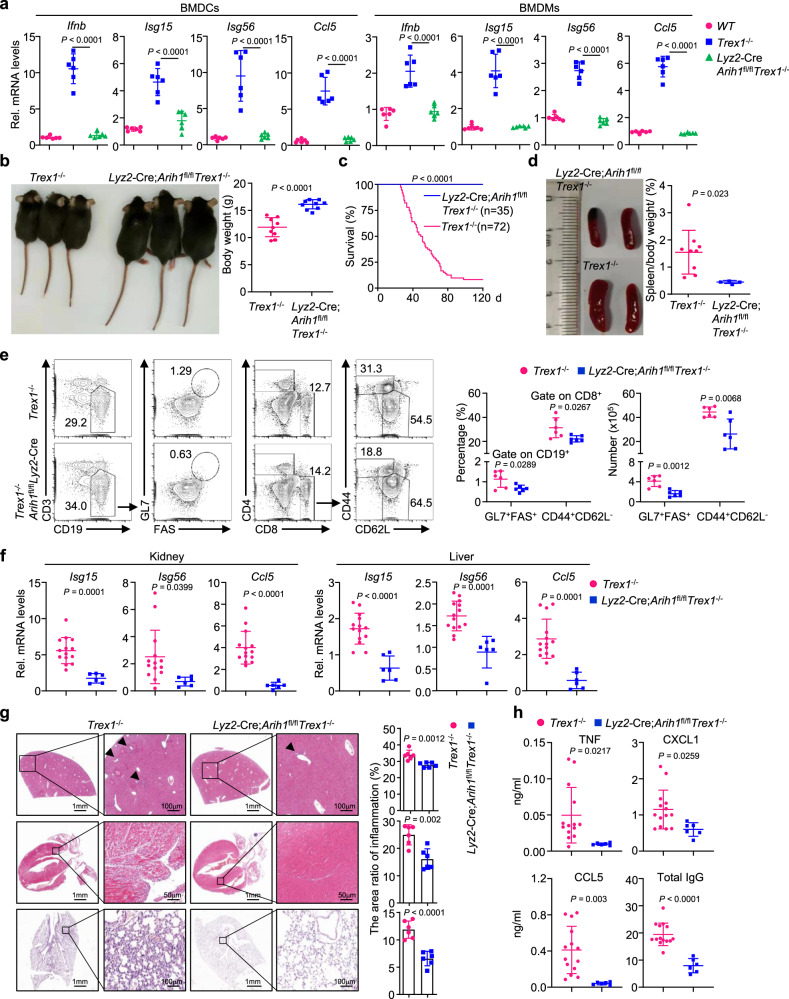
Myeloid deletion of ARIH1 rescues *Trex1*^−/−^ mice from autoimmune lethality. **a** qRT-PCR analysis of *Ifnb*, *Isg15*, *Isg56*, and *Ccl5* mRNA in wild type (*n* = 6), *Trex1*^−/−^ (*n* = 6) and *Lyz2*-Cre;*Arih1*^fl/fl^*Trex1*^−/−^ (*n* = 6) BMDCs (left graphs) or BMDMs (right graphs). **b** Representative image (left image) and body weight (right graph) of *Trex1*^−/−^ (*n* = 11) and *Lyz2*-Cre;*Arih1*^fl/fl^*Trex1*^−/−^ mice (*n* = 9) (5-week old). **c** Survival (Kaplan–Meier curve) of *Trex1*^−/−^ (*n* = 72) and *Lyz2*-Cre;*Arih1*^fl/fl^*Trex1*^−/−^ (*n* = 35) mice. **d** Representative image (left image) of spleen and percentage of spleen/body weight (right graph) of *Trex1*^−/−^ (*n* = 9) and *Lyz2*-Cre;*Arih1*^fl/fl^*Trex1*^−/−^ (*n* = 4) mice (5-week old). **e** Flow cytometry analysis of splenocytes from *Trex1*^−/−^ (*n* = 6) and *Lyz2*-Cre;*Arih1*^fl/fl^*Trex1*^−/−^ mice (*n* = 6) (5-week old) stained with fluorescence-conjugated antibodies against the indicated surface molecules. **f** qRT-PCR analysis of *Isg15*, *Isg56*, and *Ccl5* mRNA in kidney (left graphs) or liver (right graphs) from *Trex1*^−/−^ (*n* = 14) and *Lyz2*-Cre;*Arih1*^fl/fl^*Trex1*^−/−^ (*n* = 6) mice (5-week old). **g** Representative images and quantification of immune cell infiltration of HE-stained liver, heart and lung sections from *Trex1*^−/−^ (*n* = 6) and *Lyz2*-Cre;*Arih1*^fl/fl^*Trex1*^−/−^ (*n* = 6) mice (5-week old). Arrows indicate infiltrated immune cells. **h** ELISA analysis of TNF, CXCL1, CCL5, and total IgG in the sera of *Trex1*^−/−^ (*n* = 14) and *Lyz2*-Cre;*Arih1*^fl/fl^*Trex1*^−/−^ mice (*n* = 6) (5-week old). Data are representative of two independent experiments (**a**, **d**–**h**). Graphs show mean ± S.D. (*n* = 6 for (**a**, **e**, **g**), or *n* = 9 for (**b**), or *n* = 9 and 4 for (**d**), or *n* = 14 and 6 for (**f**, **h**), biologically independent experiments). Statistical significance was determined using two-tailed Student’s *t* tests in (**b**, **d**–**h**), or one-way ANOVA in a. Source data are provided as a Source Data file.

In addition to viral DNA, self-DNA accumulated in cytosol activates cGAS to induce autoimmunity. Knockout or dysfunction of TREX1 leads to the accumulation of DNA in the cytosol and causes severe autoimmune symptoms that can be eliminated by ablation of cGAS^16,53^. Since ARIH1 facilitated the activation and oligomerization of cGAS after cytoplasmic DNA challenge, we examined whether knockout of ARIH1 inhibited the autoimmune phenotypes in TREX1-deficient cells and mice. We obtained the *Lyz2*-Cre;*Arih1*^fl/fl^ *Trex1*^−/−^ mice by crossing *Lyz2*-Cre *Arih1*^fl/fl^ mice with *Trex1*^+/−^ mice^25^ and isolated the bone marrow cells from the mice for in vitro culture. Results from qRT-PCR analyses showed that the expression levels of *Ifnb*, *Isg15*, *Isg56*, and *Ccl5* in BMDCs and BMDMs from *Lyz2*-Cre;*Arih1*^fl/fl^*Trex1*^−/−^ mice were significantly lower than those from the *Trex1*^−/−^ counterparts, and were comparable to those from the wild-type counterparts (Fig. 5a). Consistently, the cGAMP activity was significantly decreased in *Lyz2*-Cre;*Arih1*^fl/fl^*Trex1*^−/−^ BMDCs and BMDMs compared to *Trex1*^−/−^ counterparts (Supplementary Fig. 4g), suggesting that deletion of ARIH1 in *Trex1*^−/−^ cells normalizes the basal expression of IFN-stimulated genes and the basal production of cGAMP.

In addition, knockout of ARIH1 in myeloid cells in *Trex1*^−/−^ background rescued the developmental retardation of *Trex1*^−/−^ mice (Fig. 5b). Expectedly, knockout of ARIH1 in myeloid cells fully rescued the autoimmune lethality caused by TREX1 deficiency, suggesting that ARIH1 in myeloid cells sufficiently supports autoimmunity caused by dysfunction of TREX1 (Fig. 5c). In this context, it has been shown that TREX1 deficiency in DCs or Cx3cr1^+^ tissue macrophages is sufficient to trigger spontaneous IFN responses and autoimmune lethal phenotypes^54^. We next analyzed the autoimmune phenotypes in various organs or tissues of *Trex1*^−/−^ mice and *Lyz2*-Cre;*Arih1*^fl/fl^*Trex1*^−/−^ mice. Firstly, we observed that the sizes of spleens were significantly smaller, and the percentages and the numbers of GL7^+^FAS^+^ B cells and CD8^+^CD44^+^CD62L^−^ T cells were substantially less in the spleens from *Lyz2*-Cre;*Arih1*^fl/fl^*Trex1*^−/−^ mice than those from *Trex1*^−/−^ mice (Fig. 5d, e). Secondly, the expression levels of *Isg15*, *Isg56*, and *Ccl5* in the kidney and liver of *Lyz2*-Cre;*Arih1*^fl/fl^*Trex1*^−/−^ mice were significantly lower than those of *Trex1*^−/−^ mice (Fig. 5f). Thirdly, the infiltration of immune cells into the liver, heart or lung of *Lyz2*-Cre;*Arih1*^fl/fl^*Trex1*^−/−^ mice were substantially compromised compared to *Trex1*^−/−^ mice as revealed by HE-staining analysis (Fig. 5g). Lastly, the levels of TNF, CXCL1, CCL5, and total IgG were significantly lower in the sera from *Lyz2*-Cre;*Arih1*^fl/fl^*Trex1*^−/−^ mice than those from *Trex1*^−/−^ mice (Fig. 5h). Taken together, these data demonstrate that ARIH1 promotes self-DNA-triggered autoimmunity.

### ARIH1 catalyzes mono-ISGylation of cGAS after HSV-1 infection

**Fig. 6:**
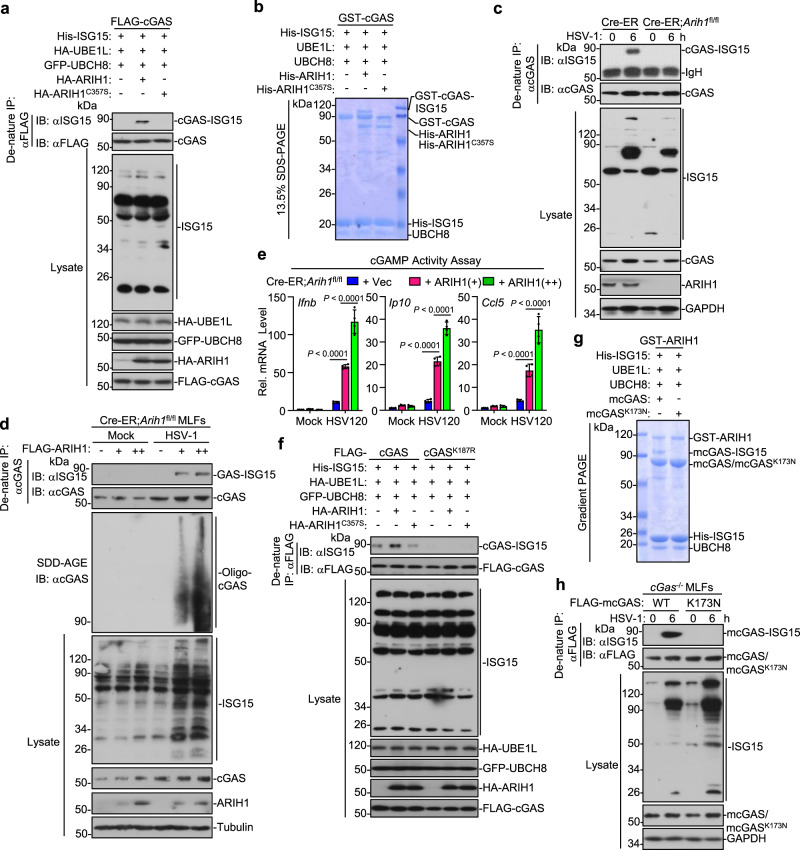
ARIH1 catalyzes mono-ISGylation of cGAS at Lys187. **a** Denature-IP (with anti-FLAG) and immunoblot analysis (with anti-FLAG, anti-HA, anti-GFP, or anti-ISG15) of HEK293 cells transfected with plasmids encoding FLAG-cGAS, HA-UBE1L, His-ISG15, and GFP-UBCH8 together with an empty vector, HA-ARIH1 or HA-ARIH1^C357S^ for 24 h. **b** GST-cGAS was incubated with UBE1L, UBCH8, His-ISG15, and His-ARIH1, or His-ARIH1^C357S^ in the reaction buffer for 2 h and subject to 13.5% SDS-PAGE followed by Coomassie brilliant blue staining. **c** Denature-IP (with anti-cGAS) and immunoblot analysis (with anti-cGAS, anti-ARIH1, anti-GAPDH, or anti-ISG15) of Cre-ER and Cre-ER;*Arih1*^fl/fl^ MLFs that were treated with 4-OHT (1 μM) for 3 days followed by HSV-1 infection for 0–6 h. **d** Denatire-IP (with anti-cGAS) and immunoblot analysis (with anti-cGAS, anti-ISG15, anti-ARIH1, or anti-Tubulin) with SDS-PAGE or SDD-AGE of 4OHT (1 μM, 3 days)-treated Cre-ER and Cre-ER;*Arih1*^fl/fl^ MLFs that were reconstituted with different amounts of Lenti-FLAG-ARIH1 followed by infection with HSV-1 for 6 h. **e** cGAMP activity assay of cells treated as in (**d**) that were transfected with HSV120 (1 μg) for 6 h. **f** Denature-IP (with anti-FLAG) and immunoblot analysis (with anti-FLAG, anti-HA, anti-GFP, or anti-ISG15) of HEK293 cells transfected with plasmids encoding FLAG-cGAS, FLAG-cGAS^K187R^, HA-UBE1L, His-ISG15, and GFP-UBCH8 together with an empty vector, HA-ARIH1 or HA-ARIH1^C357S^ for 24 h. **g** mcGAS or mcGAS^K173N^ was incubated with UBE1L, UBCH8, His-ISG15, and GST-ARIH1 in the reaction buffer for 2 h and subject to gradient SDS-PAGE followed by Coomassie brilliant blue staining. **h** Denature-IP (with anti-FLAG) and immunoblot analysis (with anti-FLAG, anti-ARIH1, anti-GAPDH, or anti-ISG15) of *cGas*^−/−^ MEFs reconstituted with FLAG-mcGAS or mcGAS^K173N^ followed by HSV-1 infection for 0–6 h. Data are representative of three independent experiments (**a**–**h**) Graphs show mean ± S.D. (*n* = 4 for (**e**), biologically independent experiments). Statistical significance was determined by one-way ANOVA in (**e**) Source data are provided as a Source Data file.

It has been reported that ARIH1 is a component of the human cullin-RING E3 ligases (CRLs) and could efficiently catalyze mono-ubiquitination of substrates dependently on the NEDDylation of CRL^45,46^. Considering that ARIH1 interacts with cGAS and promotes cGAS activation dependently on its enzymatic activity, we examined whether ARIH1 catalyzed ubiquitination of cGAS. Unexpectedly, ARIH1 did not induce obvious ubiquitination of cGAS (Supplementary Fig. 5a). Previous studies have reported that cGAS undergoes NEDDylation and SUMOylation^26,27^. However, neither ARIH1 nor ARIH1^C357S^ affected the NEDDylation or SUMOylation in HEK293 cells in our transient transfection and denature-IP assays (Supplementary Fig. 5b, c). In addition to serving as a ubiquitin ligase, ARIH1 has been suggested as an ISG15 ligase to catalyze ISGylation of 4EHP, an mRNA 5′ cap structure-binding protein^43^. Interestingly, we found that ARIH1 but not ARIH1^C357S^ catalyzed robust mono-ISGylation of cGAS in HEK293 cells (Fig. 6a and Supplementary Fig. 5d). Moreover, cGAS was basally ISGylated in the presence of ISGylation machinery including UBE1L, UBCH8, and ISG15, which was substantially impaired by knockdown of ARIH1, indicating that endogenous ARIH1 mediates mono-ISGylation of cGAS in HEK293 cells (Supplementary Fig. 5e). As a component of CRLs, the ubiquitin ligase activity is activated by NEDD8 activating enzyme (NAE)-mediated NEDDylation on cullin proteins that is inhibited by the compound MLN4924^46^. Interestingly, however, MLN4924 treatment did not affect ARIH1-mediated mono-ISGylation of cGAS in HEK293 cells (Supplementary Fig. 5f), supporting the notion that the ISG15 ligase activity but not the ubiquitin ligase activity of ARIH1 catalyzes the modification of cGAS. Results from in vitro ISGylation assays further confirmed that ARIH1 but not ARIH1^C357S^ catalyzed mono-ISGylation of cGAS (Fig. 6b). In contrast, HSV-1 infection induced mono-ISGylaiton of cGAS which was completely diminished by knockout of ARIH1 (Fig. 6c). In addition, transfection of ARIH1 into 4OHT-treated Cre-ER;*Arih1*^fl/fl^ MLFs increased the mono-ISGylation of cGAS after HSV-1 infection and promoted cGAMP activity after transfection of DNA120 in a dose-dependent manner (Fig. 6d, e). These data together suggest that ARIH1 serves as a primary ISG15 ligase E3 for mono-ISGylation of cGAS after HSV-1 infection.

### ARIH1 catalyzes mono-ISGylation of hcGAS at K187 and mcGAS at K173

We next identified the lysine residue(s) in cGAS to which ISG15 was attached by ARIH1 through liquid chromatography-mass spectrometry (LC-MS) assays (Supplementary Fig. 6a). These efforts led to the identification of the peptide “DDISTAAGMVK(GG)GVVDHLLLR”, indicating that the Lys187 of cGAS was modified by the Gly-Gly peptide (Supplementary Fig. 6a). We generated FLAG-cGAS^K187R^ mutant and found that mutation of Lys187 to arginine abolished ARIH1-mediated mono-ISGylation of cGAS in HEK293 cells or in vitro (Fig. 6f, and Supplementary Fig. 6b). In addition, HSV-1 induced mono-ISGylation of cGAS but not cGAS^K187R^ when reconstituted into *cGas*^−/−^ MEFs (Supplementary Fig. 6c), indicating that Lys187 is the ISGylated site of cGAS after HSV-1 infection. Sequence alignment analysis indicated that the Lys173 residue of murine cGAS exhibited homology with the Lys187 residue of human cGAS (Supplementary Fig. 6d). Consistent with this notion, ARIH1 catalyzed mono-ISGylation of mcGAS but not mcGAS^K173N^ in HEK293 cells and in vitro (Fig. 6g and Supplementary Fig. 6d). In addition, HSV-1 induced mono-ISGylation of mcGAS but not mcGAS^K173N^ when reconstituted into *cGas*^−/−^ MEFs (Fig. 6h). These data together demonstrate that ARIH1 catalyzes mono-ISGylation on Lys187 of hcGAS and Lys173 of mcGAS, respectively.

### Lys187 of cGAS restricts its optimal activation and oligomerization

**Fig. 7:**
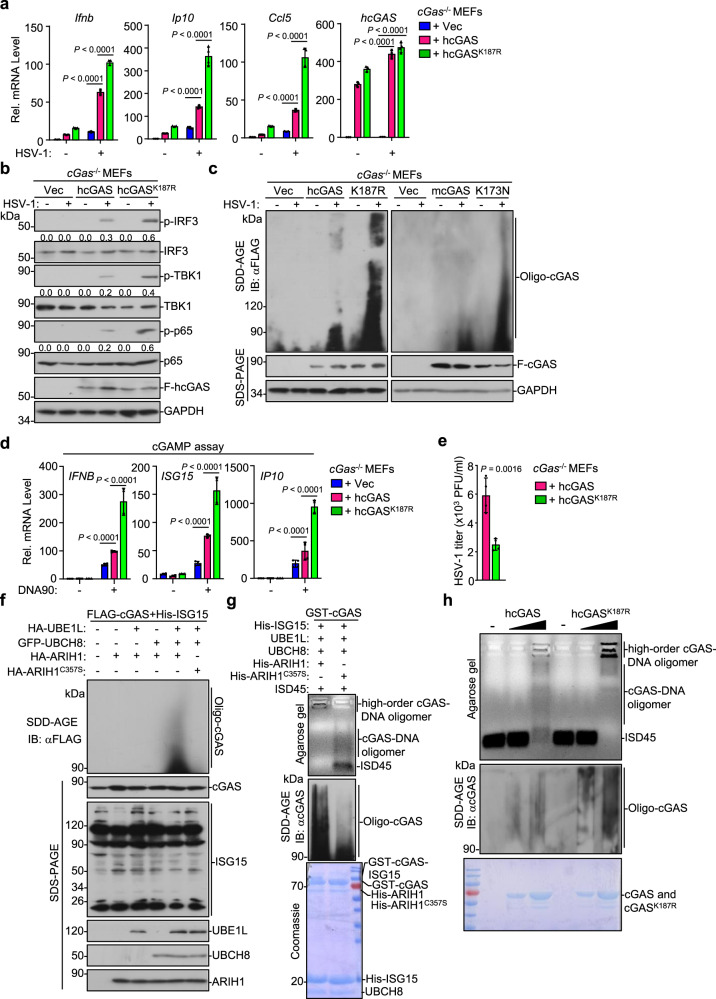
Mono-ISGylation of cGAS relieves K187-mediated inhibition of cGAS oligomerization. **a** qRT-PCR analysis of *Ifnb*, *Ip10*, *Ccl5*, and *hcGAS* mRNA in *cGas*^−/−^ MEFs reconstituted with an empty vector (Vec), hcGAS, or hcGAS^K187R^ followed by HSV-1 infection for 0–8 h. **b** Immunoblot analysis of total and phosphorylated (p−) p65, IRF3, and TBK1, FLAG-hcGAS, and GAPDH in *cGas*^−/−^ MEFs reconstituted with an empty vector (Vec), hcGAS, or hcGAS^K187R^ followed by HSV-1 infection for 0–6 h. **c** Immunoblot analysis of cGAS oligomerization in *cGas*^−/−^ MEFs reconstituted with an empty vector (Vec), FLAG-tagged hcGAS, hcGAS^K187R^, mcGAS, or mcGAS^K173N^ followed by HSV-1 infection for 0–6 h. **d** *cGas*^−/−^ MEFs reconstituted with an empty vector (Vec), hcGAS, or hcGAS^K187R^ were transfected with DNA90 (1 μg) for 4 h. The cell homogenztes were used for cGAMP assay with HFFs by qRT-PCR analysis. **e** Plaque assay of HSV-1 titers in the supernatants of *cGas*^−/−^ MEFs reconstituted with an hcGAS or hcGAS^K187R^ infected with HSV-1 (MOI = 0.5) for 12 h. **f** Immunoblot analysis of cGAS oligomerization and the expression levels of FLAG-cGAS, His-ISG15, HA-UBE1L, GFP-UBCH8, HA-ARIH1, and HA-ARIH1^C357S^ in HEK293 cells that were transfected with FLAG-cGAS and His-ISG15 together with the indicated plasmids for 24 h. **g** Agarose gel electrophoresis (upper) and immunoblot analysis (with anti-cGAS, middle) of the mixtures containing GST-cGAS, UBE1L, UBCH8, His-ISG15, and ARIH1 or ARIH1^C357S^ that reacted for 2 h in the presence of ATP (5 mM) (bottom) followed by incubation with ISD45 (2 μ mol) for 30 min. **h** Agarose gel electrophoresis (upper) and immunoblot analysis (with anti-cGAS, middle) of wild-type cGAS or cGAS^K187R^ (0, 3, 9 μg, respectively, bottom) incubated with ISD45 (2 μ mol) for 30 min. Data are representative of three independent experiments (**a**–**h**). Graphs show mean ± S.D. (*n* = 4) for (**a**, **d**, **e**), biologically independent experiments). Statistical significance was determined using two-tailed Student’s *t* tests in (**e**) or one-way ANOVA in (**a**, **d**) The quantitative anaylsis is derive from the same experiment and that blots were processed in parallel in (**b**) Source data are provided as a Source Data file.

We next investigated whether and how mono-ISGylation of cGAS affected cGAS-mediated innate immune response against DNA viruses. The *cGas*^−/−^ MEFs were stably transfected with an empty vector, cGAS or cGAS^K187R^ followed by HSV-1 infection and various analyses. Interestingly, however, we found that reconstituted cGAS^K187R^ significantly promotes HSV-1-induced expression of *Ifnb*, *Ip10*, and *Ccl5* compared to wild-type cGAS (Fig. 7a). Similarly, reconstitution of mcGAS^K173N^ into *cGas*^−/−^ MEFs led to higher levels of *Ifnb*, *Ip10* and *Cxcl1* than did wild-type mcGAS after HSV-1 infection (Supplementary Fig. 6e). In addition, reconstitution of cGAS^K187R^ into *cGas*^−/−^ MEFs promoted HSV-1-induced phosphorylation of IRF3, TBK1 and p65 more profoundly than did reconstitution of wild-type cGAS (Fig. 7b). Consistent with these observations, the *cGas*^−/−^ MEFs reconstituted with cGAS^K187R^ produced higher levels of cGAMP after transfection of DNA ligands and exhibited more intensive oligomerization and resulted in lower HSV-1 titers in the supernatants after HSV-1 infection than those reconstituted with wild-type cGAS (Fig. 7c–e), indicating that loss of mono-ISGylation of cGAS at K187 potentiates HSV-1-triggered signaling.

The above data suggested that ARIH1 catalyzed mono-ISGylation of cGAS at K187 and mcGAS at K173 to promote antiviral immune responses, whereas cGAS^K187R^ and mcGAS^K173N^ that lost the mono-ISGylation also promoted cellular antiviral immunity, which prompted us to hypothesize that K187 of hcGAS and K173 of mcGAS might inhibit its optimal activity. To test this hypothesis, we further mutated K187 of cGAS into different amino-acid residues including polar uncharged serine (S) and glycine (G), polar negative-charged aspartic acid (D), or nonpolar phenylalanine (F). Results from luciferase reporter assays showed that all the mutants activated the IFN-β promoter and NF-κB reporter more potently than wild-type cGAS in HEK293 cells that were stably transfected MITA (Supplementary Fig. 7a). In addition, we stably transfected these mutants into *cGas*^−/−^ MEFs followed by HSV-1 infection and various analyses. Results from qRT-PCR analyses suggested that the expression levels of *Ip10*, *Ccl5* and *Tnf* in *cGas*^−/−^ MEFs reconstituted with cGAS^K187S^, cGAS^K187D^, cGAS^K187G^, or cGAS^K187F^ were significantly higher than those in *cGas*^−/−^ MEFs reconstituted with wild-type cGAS (Supplementary Fig. 7b). Consistently, HSV-1-induced oligomerization of cGAS was substantially potentiated when the K187 was mutated into S, D, G, F, or R (Fig. 7c and Supplementary Fig. 7c) and when the K173 of mcGAS was mutated into N (Fig. 7c). These data indicate that K187 of cGAS and K173 of mcGAS inhibit the optimal activation and oligomerization of cGAS after HSV-1 infection or cytoplasmic DNA challenge.

### Mono-ISGylation of cGAS relieves K187-mediated inhibition of cGAS oligomerization

We further analyzed the effects of ARIH1-mediated mono-ISGylation of cGAS on the oligomerization of cGAS in cells and found that ARIH1 but not ARIH1^C357S^ induced oligomerization of cGAS in HEK293 cells that were transfected with cGAS and the ISGylation machinery (Fig. 7f). Next, we examined whether ISGylation of cGAS affected DNA-induced oligomerization of cGAS in vitro. GST-cGAS was incubated with the UBE1L, UBCH8, His-ISG15 and His-ARIH1 or His-ARIH1^C357S^ followed by incubation with ISD45. The cGAS oligomers were determined by monitoring the migration of DNA in agarose gels and the migration of cGAS in SDD-AGE gels. As expected, ISD45 efficiently induced slowly migrating GST-cGAS-ISD45 complexes and cGAS oligomerization in the ARIH1^C357S^-containing reaction mixture where cGAS was not ISGylated (Fig. 7g). In contrast, the migration of GST-cGAS-ISD45 was substantially slowed, and notably most of the cGAS-DNA complexes were in the loading well and could not migrate into the agarose gel in the ARIH1-containing reaction mixture where cGAS was mono-ISGylated (Fig. 7g), supporting the notion that mono-ISGylation promotes oligomerization of cGAS. Furthermore, we observed that ISD45 induced the oligomerization of cGAS^K187R^ more potently than cGAS in vitro and that the cGAS^K187R^-ISG15 complexes migrated much slower than the cGAS-ISG15 complexes in vitro (Fig. 7h). Taken together, these data indicate that K187 of cGAS inhibits DNA-induced oligomerization of cGAS, which is relieved by ARIH1-mediated mono-ISGylation on K187 of cGAS.

## Discussion

The activity and stability of the cytoplasmic DNA sensor cGAS are precisely regulated by various posttranslational modifications to initiate antiviral immunity and avoid harmful autoimmunity^2,55^. In this study, we have demonstrated that the E3 ubiquitin ligase ARIH1 catalyzes mono-ISGylation of cGAS and promotes oligomerization and activation of cGAS after HSV-1 infection or DNA challenge, therefore promoting the expression of downstream type I IFNs and proinflammatory cytokines (Supplementary Fig. 7d). In support of this notion, we found that (i) ARIH1 interacts with cGAS and catalyzes mono-ISGylation on K187 of hcGAS and K173 of mcGas in cells or in vitro dependently on its ligase activity; (ii) knockdown or knockout of ARIH1 impaired HSV-1- and cytoplasmic DNA- but not cGAMP-induced phosphorylation of IRF3, TBK1 and p65 and production of type I IFNs and proinflammatory cytokines, inhibited HSV-1-induced oligomerization of cGAS, and attenuated cytoplasmic DNA-induced generation of cGAMP; and (iii) ARIH1-deficient mice and cells exhibited increased susceptibility to HSV-1 infection compared to the control counterparts and deletion of ARIH1 in myeloid cells rescued the autoimmune phenotypes in *Trex1*^−/−^ mice. Collectively, these findings reveal a previously uncharacterized posttranslational modification of cGAS that promotes optimal activation of cGAS and cGAS-mediated antiviral immunity and autoimmunity.

ISG15 is promptly upregulated by viral infection and type I IFNs^30^. Previous studies have shown that *Isg15*^−/−^ mice and *Ube1l*^-/-^ mice are hypersensitive to HSV-1 infection compared to wild-type mice^33^, while USP18^C61A/C61A^ mice in which ISGylation is accumulated are resistant to Vaccinia virus infection^35^, indicating a protective role of ISG15 and ISG15 conjugation in immune defense against DNA viruses. However, the key ISGylated targets in host remain to be characterized. In addition, ISG15 is one of the immediate-early genes induced by viral infections independently of type I IFNs and its roles in defense against viruses at an early stage are not fully understood^56–58^. We found that ARIH1 catalyzed the conjugation of ISG15 to cGAS at early time points after HSV-1 infection, which promotes its activation and oligomerization and thereby facilitates innate immune responses against DNA viruses. Consistent with this notion, knockout of ARIH1 abolished HSV-1-induced mono-ISGylation of cGAS and resulted in hypersensitivity to HSV-1 infection. Although it is currently unknown whether there exist additional ISGylated targets of ARIH1 in the context of viral infection, the available data have suggested a positive feedback loop of cGAS ISGylation by ARIH1 to promote antiviral immunity. In contrast to an antiviral role of ISG15 in mice, patients deficient in ISG15 display autoinflammatory interferonopathies as a result of the impaired accumulation of USP18 which is a potent negative regulator of type I IFN signaling^59^, indicating that a major function of human ISG15 is likely to attenuate type I IFN responses rather than to facilitate antiviral responses. However, it should be noted that ISG15-deficient patients are prone to mycobacterial disease^59^. It is well recognized that cGAS is an indispensable sensor of *Mycobacterium tuberculosis* and mediates protective immune responses against mycobacterial infections^60–62^. Therefore, the loss of mono-ISGylation of cGAS might be involved in mycobacterial disease of ISG15-deficient patients as a result of impaired activity of cGAS. It is of great interest to examine whether ARIH1-mediated mono-ISGylation of cGAS promotes immune responses against *Mycobacterium tuberculosis* infections in the future.

It has been shown that ARIH1 is a component of CRLs and activated by NEDD8-Cullin-RBX1 complex to catalyze mono-ubiquitination of CRL substrates^45,46^. Our data showed that ARIH1 catalyzed mono-ISGylation of cGAS independently of CRLs. Firstly, treatment of MLN4924 that inhibits NEDD8 conjugation to Cullin proteins and inactivates CRLs did not affect ARIH1-mediated mono-ISGylation of cGAS in HEK293 cells. Secondly, ARIH1 failed to increase the ubiquitination of cGAS in cells or in vitro. Thirdly, ARIH1 but not the enzymatic inactive mutant ARIH1^C357S^ directly catalyzed mono-ISGylation of cGAS in the presence of UBE1L and UBCH8 as required for ISGylation and in the absence of CRLs and UBCH7 as required for mono-ubiquitination^46,47^. These data have demonstrated that ARIH1 functions as an E3 ISG15 ligase for optimal activation of cGAS in the context of antiviral immunity and autoimmunity. Structural analyses suggest that the ARIH1 catalytic site (Cys357) is masked by its C-terminal Ariadne domain and that NEDD8-Cullin-RBX1 complex binds to ARIH1 and leads to conformational change and exposure of the active site of ARIH1^46^. How the catalytic site Cys357 of ARIH1 was exposed for ISGylation during HSV-1 infection remained to be elucidated. One of the possibilities might be that the association of cGAS facilitates the conformational change as does the NEDD8-Cullin-RBX1 complex, as it was noted that the C-terminal Ariadne domain of ARIH1 interacted with cGAS in co-immunoprecipitation assays. A second possibility was that the E2 UBCH8 was required and sufficient for such a conformational change of ARIH1, which was supported by the observation that ARIH1 catalyzed ISGylation only in the presence of UBCH8^43^. A third explanation for this was that the ISG15 ligase activity of ARIH1 might be regulated in a different manner. In this context, deletion of the C-terminal Ariadne domain of ARIH1 impairs its ability to catalyze ISGylation of another substrate 4EHP^43^. Further structural studies with the purified ISGylation system together with substrates would help to understand the mechanisms for ARIH1 activation as an E3 ISG15 ligase.

We have provided several lines of evidence that ISG15 was targeted on Lys187 of hcGAS and Lys173 of mcGAS. Analyses of MS data suggested that there was a Gly-Gly modification on K187 of cGAS only when cGAS was co-transfected and incubated with ARIH1, ISG15, UBE1L, and UBCH8 in HEK293 cells. Mutation of K187 or K173 completely abolished ARIH1-mediated ISGylation of hcGAS or mcGAS in vitro or in cells after HSV-1 infection, respectively. Structural analysis has suggested a DNA binding site (A site) at aa176-195 of hcGAS and aa147-181 of mcGAS in which K187 and K173 are located, respectively^5,7,8,10^. However, mutation of multiple positive charged amino-acid residues in these regions into Ala has minimal effect on the DNA binding activity of cGAS, indicating that site A plays a minor role in regard of activating cGAS^8,10^. In contrast, mutation of K187 and L195 into Asn and Arg enhances the DNA binding and oligomerization of cGAS in vitro^9^. In our study, we found that mutation of hcGAS K187 into R, S, D, G, or F and mutation of mcGAS K173 into N substantially enhanced the oligomerization and activity of cGAS in cells after HSV-1 infection and in vitro in the presence of DNA. These data strongly indicate that K187 exerts an inhibitory effect on the activation and oligomerization of cGAS. Consequently, the mono-ISGylation on K187 of hcGAS and K173 of mcGAS blocked their inhibitory effects, thereby promoting the activation and oligomerization of cGAS in cells and in vitro. So far we do not know whether and how such a modification affects the DNA binding to cGAS, as we failed to purify enough amounts of ISGylated cGAS for in vitro DNA binding assays. It should be noted that in all the cGAS structures, cGAS proteins were crystallized as truncated forms that only include NTase and Mab21 domains. It is conceivable that additional regulatory machinery exists to regulate the DNA binding, oligomerization, and activation of full-length cGAS in cells and in vitro. In this context, it has been shown that the N terminus of hcGAS binds to DNA and facilitates the optimal activation of cGAS^63^. A comprehensive understanding of the mechanisms by which ISGylation of cGAS on K187 promotes activation and oligomerization of cGAS depends on more detailed structural and functional analyses of full-length cGAS.

Recent studies have revealed a suppressive role of ARIH1 in tumorigenesis^49,51^. A more recent report has shown that ARIH1 catalyzes ubiquitination and degradation of PD-L1 and thereby promotes anti-tumor immunity and that the expression of ARIH1 is severely suppressed in lung adenocarcinoma biopsies^51^. It has been reported that cytoplasmic cGAS activates innate immune signaling in response to DNA damage or genome instability in tumor cells and thereby promotes the activation of CD8^+^ T cells and the efficacy of immune checkpoint blockades therapies^64–66^. It is of great interest to examine whether ARIH1 also exerts anti-tumor activity through promoting ISGylation and activation of cGAS. Collectively, our findings not only characterize a previously uncovered regulatory mechanism of cGAS activation but also provide potential targets for viral infection-caused diseases and autoimmune disorders.

## Methods

### Mice

The *Arih1*^fl/+^ mice were generated by GemPharmatech Co. Ltd through CRISPR/Cas9-mediated gene editing. In brief, guide RNAs (5′-GGCAGGAGCAGGCGAGCCCT-3′ and 5′-AAGTAAGTGATATAGCCCCC-3′) were obtained through in vitro transcription and purification. The gRNAs were incubated with purified Cas9 protein and injected into the fertilized eggs (at the one-cell stage) together with the targeting vector with two loxp sites flanking the exon 3 of the *Arih1* gene. The injected fertilized eggs were cultured to the two-cell stage followed by transplantation into pseudopregnant mice. The targeted genomes of F0 mice were amplified by PCR and sequenced and the chimeras were crossed with wild-type C57BL/6 mice to obtain F1 *Arih1*^fl/+^ mice. Southern blot analysis was conducted with the tail DNA from F1 mice to confirm correct recombination and exclude random insertions of the targeting vector. *Lyz2*-Cre mice (B6/JNju-Lyz2^em1Cin(iCre)^/Nju, stock number: T003822) were purchased from the Nanjing Biomedical Research Institute of Nanjing University. *Trex1*^+/−^ mice were described previously^25^. Cre-ER mice (B6.129-Gt(ROSA)26Sor^tm1(cre/ERT2)Tyj^, stock number: 008463) were from the Jackson Laboratory and kindly provided by Dr. Chen Dong (Tsinghua University). *Arih1*^fl/+^ mice were crossed with Cre-ER, *Lyz*2-Cre, or *Trex1*^+/−^ mice to obtain Cre-ER;*Arih1*^fl/fl^, *Lyz2*-Cre;*Arih1*^fl/fl^, and Lyz2-Cre;*Arih1*^fl/fl^*Trex1*^−/−^ mice, respectively. All genetic models were on the C57BL/6 background. Mice including both sexes, between the ages of 5–8 weeks were used for all described experiments. 5-week old, gender-matched *Trex1*^−/−^ and *Lyz2*-Cre;Arih1^fl/fl^*Trex1*^−/−^ mice were used for experiments. 6 to 8-week old, gender-matched mice were used for all remaining experiments. Mice genotypes were determined by PCR analysis of tail DNA, and the genotyping primers are as follows (wild-type *Arih1* allele is of 280 bp; floxed allele is of 378 bp; positive Cre-ER is of 825 bp; positive *Lyz2*-Cre is of 1413 bp; positive *Trex1* knockout allele is of 200 bp): *Arih1* forward: 5′-TGCTAAGATACTTTAGACTGGGCC-3′, reverse: 5′-TGTTATCAGGAAATGGTGTACCAAG-3′; Cre-ER forward: 5′-AAAGTCGCTCTGAGTTGTTAT-3′, reverse: 5′-CCTGATCCTGGCAATTTCG-3′; *Lyz2*-Cre forward: 5′-AGTGCTGAAGTCCATAGATCGG-3′, reverse: 5′- GTCACTCACTGCTCCCCTGT-3′; *Trex1* forward: 5′-AGGCAAATAAGTAGTGGA-3′, reverse: 5′-TCTCACTGGCCCCAGGGCTAC-3′.

### Tamoxifen-mediated deletion of ARIH1 in mice and in cells

To achieve conditional knockout of *Arih1*, 8–10-week-old Cre-ER and Cre-ER;*Arih1*^fl/fl^ mice were injected intraperitoneally with tamoxifen (80 mg per kg body weight, dissolved in corn oil) (Sigma, #T5648) for 5 successive days. After 5 days rest, mice were either euthanized to test the knockout efficiency or infected with HSV-1. To delete ARIH1 in cultured cells, Cre-ER and Cre-ER;*Arih1*^fl/fl^ cells were treated with 4-hydroxytamoxifen (4-OHT, 1 μM) (Sigma, H6278) for 3 days. Cells were then re-seeded into culture dishes or plated in 4-OHT-free medium and rested for 24 h followed by infection with HSV-1, SeV or transfection with dsDNA, poly(I:C) or cGAMP.

### Reagents, antibodies, and constructs

Poly(I:C) and dsDNA were described previously^67^. MLN4924 (Pevonedistat, HY-70062) was purchased from MedChemExpress. H129-G4 was previously described and kindly provided by Dr. Min-Hua Luo (Wuhan Institute of Virology, Chinese Academy of Sciences)^68^. Recombinant mouse GM-CSF (500-P65) and mouse M-CSF (500-P62G) were purchased from PeproTech.). Information on the antibodies used in this study is included in Supplementary Table 1. Various reporter plasmids and Mammalian expression plasmids for SUMO1/2/3 and UBC9 were kindly provided by Drs. Ming-Ming Hu and Hong-Bing Shu^26^. Mammalian expression plasmids for NEDD8 and NEDD8ΔGG were kindly provided by Dr. Ling-Qiang Zhang (Beijing Institute of Lifeomics)^69^. The cDNAs encoding human E3 ligases were amplified and cloned into the pGADT7 vectors^48^. The plasmids for cGAS, MAVS, MITA, TBK1, IRF3, RIG-I, ubiquitin were previously described^67,70,71^. Mammalian expression plasmids for ARIH1, ARIH1 mutants or truncations, cGAS mutants or truncations, ISG15, UBE1L, and UBCH8 were constructed by standard molecular biology techniques.

### Yeast two-hybrid assays

The pGBT9-cGAS vector and the individual pGADT7 vectors encoding E3s were transformed into AH109 competent cells which were grown on the Trp^−^Leu^−^ medium plates at 30 °C for 2 days. The positive clones were transferred to the Ade^−^His^−^Trp^−^Leu^−^ medium plates and cultured at 30 °C for 3–5 days. The positive clones were recognized as potential cGAS-interacting E3s.

### Transfection and reporter gene assays

HEK293 cells were transiently transfected with firefly luciferase reporter (100 ng) and TK-Renilla luciferase reporter (20 ng) and indicated plasmids or empty vector (100 ng) using standard calcium phosphate precipitation. After 24 h, luciferase assays were performed with a dual-specific luciferase reporter kit (Promega). The activity of firefly luciferase was normalized by that of Renilla luciferase to obtain relative luciferase activity.

### Transfection of DNA ligands, poly(I:C) and cGAMP

Cells were cultured in plates or dishes for 24 h before being transfected with DNA ligands and poly(I:C) via PEI reagent (Polysciences Inc, 24765-1) according to the manufacturer’s instructions. Briefly, the ligands were diluted in serum-free DMEM (the volume of DMEM is 10% of final volume of cell culture medium) (1 μg ligands in 25 μl DMEM). PEI (1 μg/μl) was diluted in an equal volume of DMEM with a ratio of 3:1 [PEI (μl):ligands (μg)]. The PEI-containing DMEM was mixed with ligands-containing DMEM and incubated for 15 min at room temperature before adding into the cell cultures. For transfection of cGAMP, the cGAMP (1 μg/μl) and 10 × digitonin were diluted in DMEM (the volume of DMEM is 50% of the cell culture medium). The supernatants of cell cultures were removed and the cGAMP-containing DMEM was added to cell cultures. Half an hour later, the cGAMP-containing DMEM was removed and full medium was added to culture the cells for different time points followed by various assays. The oligonucleotide sequences of DNA ligands were included in Supplementary Table 2.

### Co-immunoprecipitation and immunoblot assays

Cells were collected and lysed for 15 min with 800 μL Nonidet P-40 lysis buffer (20 mM Tris-HCl, pH 7.4–7.5, 150 mM NaCl, 1 mM EDTA, 1% Nonidet P-40) containing inhibitors for protease and phosphatases (TopScience). Cell lysates (700 μl) were incubated with a control IgG or specific antibodies and protein G agarose for 2–4 h. The immunoprecipitates were washed three times by 1 ml pre-lysis buffer and subject to immunoblot analysis. The rest of lysates (100 μl) were subject to immunoblot analysis to detect the expression of target proteins. All uncropped and unprocessed scans of the blots are provided in the Source Data file.

### Semi-denaturing detergent agarose gel electrophoresis (SDD-AGE)

Cells were lysed in NP-40 lysis buffer, and the cell lysates were mixed in 1 × sample buffer (0.5 × TBE, 10% glycerol, 2% SDS, and 0.0025% bromophenol blue) and loaded onto a vertical 2% agarose gel (Bio-Rad). After electrophoresis in the running buffer (1 × TBE and 0.1% SDS) for about 2 h with a constant voltage of 100 V at 4 °C, the proteins were subject to immunoblot analysis.

### Protein purification and GST pulldown assays

The plasmids encoding GST, GST-ARIH1 and GST-cGAS or encoding His-ISG15, UBE1L, UBCH8, cGAS, cGAS^K187R^, ARIH1, and ARIH1^C357S^ were transformed into BL21(DE3) or Rosetta competent cells which were induced with IPTG (1 mM) at 16 °C at 200 rpm for 16 h. The cells were lysed in lysis buffer (20 mM Tris-HCl, 200 mM NaCl, 5% glycerol, and 0.3% Triton X-100) and the proteins were purified through affinity chromatography using a glutathione-Sepharose matrix (Transgen Biotech) followed by glutathione (10 mM in 50 mM Tris-HCl) elution (for GST-tagged proteins) or using Ni-NTA agarose followed by 300 mM imidazole elution (for His-tagged proteins) and dialysis. To obtain untagged proteins, His-tagged cGAS, ARIH1 or their mutants were incubated with His-TEV enzyme at 4^o^C overnight followed by purification by a Ni-NTA column. The untagged proteins were in the flow-through fluid and used for subsequent experiments. The recombinant proteins were saved at −80 °C until use. FLAG-cGAS proteins were expressed with TNT Quick Coupled Transcription/Translation Systems kit (Promega, Madison, WI) as the manufacturer’s instructions. For GST Pull-Down assay, the purified GST and GST-ARIH1 proteins were incubated with FLAG-cGAS at 4 °C for overnight followed by ProteinIso GST Resin (Transgen Biotech) pulldown for 2 h in PBS containing protease inhibitors. The GST resin was washed 3 times with PBS and subject to immunoblot analysis and Coomassie brilliant blue staining.

### Ni-NTA pulldown assays

Cells cultured in 6 cm plates were transfected with the indicated plasmids. Twenty-four hours after transfection, cells from each plate were collected and divided into two aliquots. One aliquot was lysed in lysis buffer and analyzed by immunoblotting to examine the expression levels of transfected plasmids. The aliquot was lysed in buffer A (6 M guanidinium-HCl, 0.1 M Na_2_HPO_4_/NaH_2_PO_4_, 10 mM Tris-Cl pH 8.0, 5 mM imidazole, and 10 mM β-mercaptoethanol), and incubated with Ni^2+^-NTA beads (QIAGEN) for 4 h at room temperature or overnight at 4 °C. The beads were washed sequentially with buffers A, B (8 M urea, 0.1 M Na_2_PO_4_/NaH_2_PO_4_, 10 mM Tris-HCl pH 8.0, 10 mM β-mercaptoethanol), and C (same as B except pH 6.3). Beads with bound proteins were incubated in SDS loading buffer and heated at 95^o^C for 10 min followed by immunoblot analysis.

### Denature-IP, ISGylation, SUMOylation, ubiquitination and NEDDylation assays

Cells were lysed in regular lysis buffer (100–200 μl) and the cell lysates were denatured at 95 °C for 5 min in the presence of 1% SDS. A portion of cell lysates (20 μl) were saved for immunoblot analysis to detect the expression of target proteins. The rest of cell lysates (80–180 μl) were diluted with 1–2 ml lysis buffer and immunoprecipitated (Denature-IP) with either anti-FLAG beads or with protein G (20 μl) plus anti-FLAG (0.5 μg) or anti-cGAS (1 μg). The immunoprecipitates were washed by three times and subject to immunoblot analysis.

### In vitro ISGylation assay

GST-ARIH1, GST-cGAS, His-ISG15, hcGAS, hcGAS^K187R^, mcGAS, mcGAS^K173N^, ARIH1, and ARIH1^C357S^ proteins were purified from bacteria. UBE1L (11990-H20B) and UBCH8 (BML-UW9135) were purchased from Sino Biological and Enzo Life Sciences, respectively. In vitro ISGylation reactions were performed at 37 ^o^C for 2 h in 20 μL volume containing His-ISG15 (5 μg), UBE1L (0.2 μg), UBCH8 (0.5 μg), ARIH1 or ARIH1^C357S^ (5 μg), cGAS or cGAS^K187R^ (5 μg), mcGAS or mcGAS^K173N^ (5 μg) in the reaction buffer (50 mM Tris-HCl pH 7.5, 5 mM Mg^2+^-ATP, and 2.5 mM DTT). The mix was fractionated by SDS-PAGE and analyzed by Coomassie brilliant blue staining.

### Analysis of In vitro oligomerization of cGAS

Purified cGAS or cGAS mutants (3-9 μg) were incubated with 2 μM 45 bp dsDNA in the reaction buffer containing 20 mM HEPES-NaOH (pH 7.8), 1 mM DTT, 75 mM KCl at 4 °C for 30 min. Reactions were separated on a 2% agarose gel in 0.5 × TBE buffer at 4 °C followed by imaging with the G:BOX system. Alternatively, the reactions were loaded to SDD-AGE followed by immunoblot analysis. To examine the effect of cGAS mono-ISGylation on its oligomerization, GST-cGAS were incubated with UBE1L, UBCH8, His-ISG15 and ARIH1 or ARIH1^C357S^ in the presence of 5 mM ATP. Subsequently, the mixtures were incubated with 45 bp dsDNA (ISD45) in reaction buffer at 4 °C for 30 min followed by electrophoresis on a 2% agarose gel in 0.5 × TBE buffer at 4 °C. Alternatively, the mixtures were loaded to SDD-AGE followed by immunoblot with anti-cGAS to determine the oligomerization of cGAS.

### qRT-PCR and ELISA

Total RNA was extracted from cells using TRIzol (Life Technologies), and the first-strand cDNA was reversed-transcribed with All-in-One cDNA Synthesis SuperMix (Aidlab Biotechnologies). Gene expression was examined with a Bio-Rad CFX Manager 3.1 by a fast two-step amplification program with 2 × SYBR Green Fast qPCR Master Mix (Yeasen Biotechnology). The value obtained for each gene was normalized to that of the gene encoding β-actin. The sequences of primers for qRT-PCR analysis were included in Supplementary Table 3. The IFN-β, IL-6, TNF (Biolegend), and CCL5 (4A Biotech) protein in the sera or cell supernatants were determined by ELISA kits from the indicated manufacturers.

### Cell culture

Bone marrow cells were isolated from femurs of *Arih1*^fl/fl^ and *Lyz2*-Cre;*Arih1*^fl/fl^ mice. The cells were cultured in DMEM containing 15% (vol/vol) FBS, 1% streptomycin–penicillin. GM-CSF (20 ng/ml, Peprotech) and M-CSF (10 ng/ml, Peprotech) were added to the bone marrow culture for differentiation of BMDCs and BMDMs, respectively. THP-1 and HEK293 cells were from the American Type Culture Collection, authenticated by STR locus analysis and tested for mycoplasma contamination. Primary MLFs were isolated from ~8–10-week-old Cre-ER and Cre-ER;*Arih1*^fl/fl^ mice. Lungs were minced and digested in calcium and magnesium free HBSS buffer supplemented with 10 mg/ml type I collagenase (Worthington) and 20 μg/ml DNase I (Sigma-Aldrich) for 3 h at 37 °C with shaking. Cell suspensions were cultured in DMEM containing 15% (vol/vol) FBS, 1% streptomycin–penicillin. Two days later, adherent fibroblasts were rinsed with PBS and treated with 4-hydroxytamoxifen (1 μM) (Sigma, H6278) for 3 days before use in experiments. *cGas*^−/−^ MEFs, NCTC clone 929 clone of strain L mouse fibroblasts (L929), and human foreskin fibroblasts cells (HFFs) were kindly provided by Drs. Ming-Ming Hu and Hong-Bing Shu (Wuhan University).

### Lentivirus-mediated gene transfer

HEK293 cells were transfected with phage-6tag-ARIH1, phage-6tag-ARIH1^C357S^, phage-6tag-cGAS, phage-6tag-cGAS^K187R^, phage-6tag-mcGAS^K173N^, or the empty vector along with the packaging vectors psPAX2 and pMD2G. The medium was changed with fresh full medium (15% FBS, 1% streptomycin–penicillin) at 8 h after transfection. The supernatants were harvested 40 h later to infect 4OHT-treated Cre-ER;*Arih1*^fl/fl^ MLFs or *cGas*^−/−^ MEFs for subsequent experiments.

### Viral infection

For qRT-PCR analysis, cells were seeded into 24-well plates (13 × 10^6^ cells per well) and infected with HSV-1 or Sev for the indicated time points. For viral replication assays, cells (1–3 × 10^6^) were infected with H129-G4 or HSV-1. One hour later, the supernatants were removed and cells were washed twice with 1 ml pre-warmed PBS followed by culture in full medium for 12 h. Viral replication was analyzed by flow cytometry, fluorescent microscopy, or qPCR analysis. For the intraperitoneal infection of mice, age- and sex- matched control and ARIH1 knockout mice were injected with HSV-1 (1 × 10^7^ PFU per mouse) and the survival of animals was monitored every day. The spleens were collected for plaque assays and qRT-PCR analysis or plaque assays at 3 days after infection. For the intracranial infection of mice, age- and sex-matched control and ARIH1 knockout mice were anesthetized by intraperitoneal injection of 1% sodium pentobarbital (w/v = 1:6), followed by intracranial injection with HSV-1 (1 × 10^6^ PFU per mouse) using a 25-μl positive displacement syringe. The needle was placed in the approximate region of the hippocampus, equidistant between the lambda and bregma, through the right parietal bone lateral to the sagittal suture. The brains were collected for HE-staining analysis at 3 days after infection.

### Plaque assays

The supernatants of BMDCs or MLFs cultures and the homogenates (or the serial dilutions) of spleens from infected mice were used to infect monolayers of Vero cells. One hour later, the supernatants or homogenates were removed and the infected Vero cells were washed with pre-warmed PBS twice followed by incubation with DMEM containing 2% methylcellulose for 48 h. The cells were fixed with 4% paraformaldehyde for 15 min and stained with 1% crystal violet for 30 min before counting the plaques.

### cGAMP activity assays

Cells (1–3 × 10^7^) transfected with dsDNA were harvested and homogenized in 1 mL sterile water by a 1 mL syringe pipetting up and down for 20 times. The homogenates were heated at 95 °C for 10 min followed by centrifuge at 40,000 × *g* (Hitachi CP100NX, P90NT-1022) for 2 h. The supernatants (900 μL) were mixed with 10 × digitonin (100 μL) and incubated with the single-layered HFF cells or L929 cells (2 × 10^5^). Half an hour later, the supernatants were removed and full medium was added to treat HFFs for 4 h followed by qPCR assays.

### shRNA

The shRNAs targeting ARIH1 were constructed by plasmid pLentiLox 3.7 and transfected using Ultra Fection2.0 (4 A Biotech) or delivered to cells using lentivirus followed by qRT-PCR or immunoblot analysis. The shRNA sequences used in this study are as follows: shARIH1#1: 5′-GAACTACCCTAACTCGTATTT-3′; shARIH1#2:

5′-GCTACCTTGAACGAGATATTT-3′

### Hematoxylin-eosin (HE) staining

Tissues from mice were fixed in 4% paraformaldehyde and embedded in paraffin blocks. The paraffin blocks were sectioned (5 μm) for HE staining (Beyotime Biotech) followed by cover-slipped. Images were acquired using an Aperio VERSA 8 (Leica) multifunctional scanner.

### Flow cytometry analysis

Single-cell suspension was resuspended in FACS buffer (PBS, 1% BSA) and blocked with anti-mouse CD16/32 antibodies for 10 min prior to staining with antibodies against surface markers (Biolgend). Flow cytometry data were acquired on a FACS Celesta or Fortesa flow cytometer, BD FACSDiVa Software v8.0.1.1, and analyzed with FlowJo software (TreeStar).

### LC-MS analysis

HEK293 cells were co-transfected with plasmids encoding ISG15, UBE1L, UBCH8 and FLAG-ARIH1 or empty vector for 24 h. Cells were then collected and lysed for immunoprecipitation (Denature-IP) with anti-FLAG beads followed by elution with 3 × FLAG peptide (0.3 mg/ml) in 1% SDC+ buffer (10 mM TCEP, 40 mM CAA, 1%SDC, 100 mM Tris-HCl). (Sigma, F4799). The eluted proteins were diluted with an equal volume of water to reduce the SDC concentration to 0.5% before being subjected to trypsin digestion and Liquid chromatography-mass spectrometry (LC-MS) analysis as previously described^72^. In brief, trypsin (1 μg) was added to the diluted elutions followed by overnight incubation at 37 °C while shaking. The reactions were quenched by TFA (final concentration, 1%) followed by centrifuge for 5 min at 12,000 × *g*. The supernantants containing peptides were transferred to a fresh tube and desalted by SDB-RPS stage tips. The desalted peptides were dissolved in MS loading buffer (0.1% formic acid), loaded onto a C18 trap column (100 μm × 20 mm, 3 μm particle size, 120 Å pore size) through an auto-sampler and then eluted into a C18 analytical column (75 μm × 250 mm, 2 μm particle size, 100 Å pore size). Mobile phase A (0.1% formic acid) and mobile phase B (90% ACN, 0.1% formic acid) were used to establish a 60 min separation gradient. A constant flow rate was set at 300 nL per min. Data was acquired using a spray voltage of 2 kV, Ion funnel RF of 40, and ion transfer tube temperature of 320 °C. For DDA mode analysis, each scan cycle consisted of one full-scan mass spectrum (Res. 60 K, scan range 350–1500 m/z, AGC 300%, IT 20 ms) followed by MS/MS events (Res. 15 K, AGC 100%, IT auto). Cycle time was set to 2 s. Isolation window was set at 1.6 Da. Dynamic exclusion time was set to 35 s. Normalized collision energy was set at 30%. For Parallel reaction monitoring, each sample was analyzed under PRM with an isolation window of 1.6 Da. In all experiments, a full mass spectrum (Res. 60 K, AGC target 300%, scan range 350–1500 m/z, IT 30 ms) was followed by up to 24 PRM scans (Res. 30 K, AGC target 300%, IT 100 ms), as triggered by a unscheduled inclusion list. PRM data were manually curated within Skyline (version 21.1.0.278).

### Ethical approval

All mice were housed in the specific pathogen-free facility (SPF), and virus infection experiments were carried out in animal biosafety level 2 (ABSL-2) facility at the Medical Research Institute of Wuhan University. Carbon dioxide was used for euthanasia. The experimental protocol adhered to the International Guiding Principles for Biomedical Involving Animals. The protocol for animal experiments was approved by the Institutional Animal Care and Use Committee of the Medical Research Institute of Wuhan University (approval number S10221020A).

### Statistical analysis

Differences between experimental and control groups were determined using two-tailed Student’s *t* test (where two groups of data were compared) or one-way ANOVA (where more than two groups of data were compared). *P* values <0.05 were considered statistically significant. For animal survival analysis, the Kaplan–Meier method was used to generate the graphs, and the survival curves were analyzed using log-rank tests.

### Reporting summary

Further information on research design is available in the Nature Research Reporting Summary linked to this article.

## Supplementary information

Supplementary Information

Reporting Summary

## Source data

Source Data

## Data Availability

All data are included in the Supplementary Information or available from the authors upon reasonable requests, as are unique reagents used in this Article. Source data are provided with this paper.
