# Supplementary material for: The E3 ubiquitin ligase ARIH1 promotes antiviral immunity and autoimmunity by inducing mono-ISGylation and oligomerization of cGAS
Source: Nat Commun. 2022 Oct 10;13:5973. doi: 10.1038/s41467-022-33671-5 (PMC9551088; doi:10.1038/s41467-022-33671-5)
Supplement: Supplementary file 1 — Supplementary Information [file 41467_2022_33671_MOESM1_ESM.pdf]

# Supplementary Information

## The E3 ubiquitin ligase ARIH1 promotes antiviral immunity and autoimmunity by inducing mono-ISGylation and oligomerization of cGAS

Xiong et al

# Supplementary Figure 1

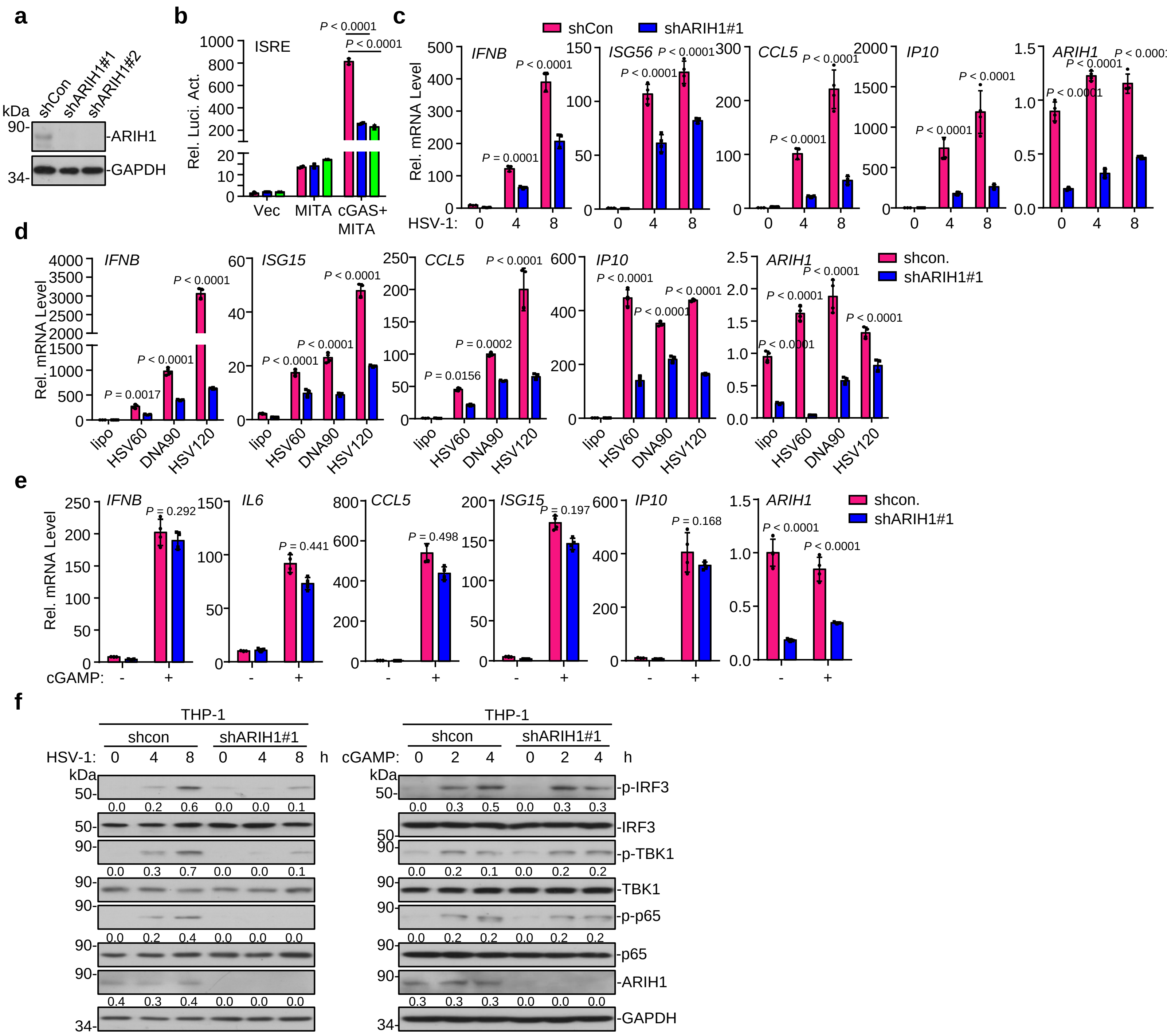


**Supplementary Figure 1 Knockdown of ARIH1 inhibits HSV-1-induced expression of downstream genes.**

(**a**) Immunoblot analysis of ARIH1 and GAPDH in HEK293 cells that were transfected with shCon, shARIH1#1 or shARIH1#2 for 36 h.

(**b**) Luciferase reporter assays analyzing ISRE promoter activity in HEK293 cells transfected with shCon, shARIH1#1 or shARIH1#2 together with the indicated plasmids for 36 h.

(**c-e**) qRT-PCR analysis of *IFNB*, *ISG56*, *CCL5*, *IP10* and *ARIH1* mRNA in THP-1 cells stably transfected with shCon or shARIH1#1 followed by infection with HSV-1 for 0-8 h **(c),** transfection with HSV60, DNA90 and HSV120 for 4 h (**d**) or transfection with cGAMP for 4 h (**e**).

(**f**) Immunoblot analysis of total and phosphorylated (p-) IRF3,TBK1 and P65, ARIH1 and GAPDH in THP-1 cells stably transfected with shCon or shARIH1#1 followed by infection with HSV-1 for 0-8 h or transfection with cGAMP for 0-4 h .

Data are representative of three (**a**, **c-e**) or two (**b**, **f**) independent experiments. Graphs show mean ± S.D. (n = 3 for **b**, or n = 4 for **c-e**, biologically independent experiments). Statistical significance was determined using two-tailed Student's *t*-test in **c-e**, or one-way ANOVA in **b**. The quantitative anaylsis is derive from the same experiment and that blots were processed in parallel in **f**. Source data are provided as a Source Data file.

# Supplementary Figure 2

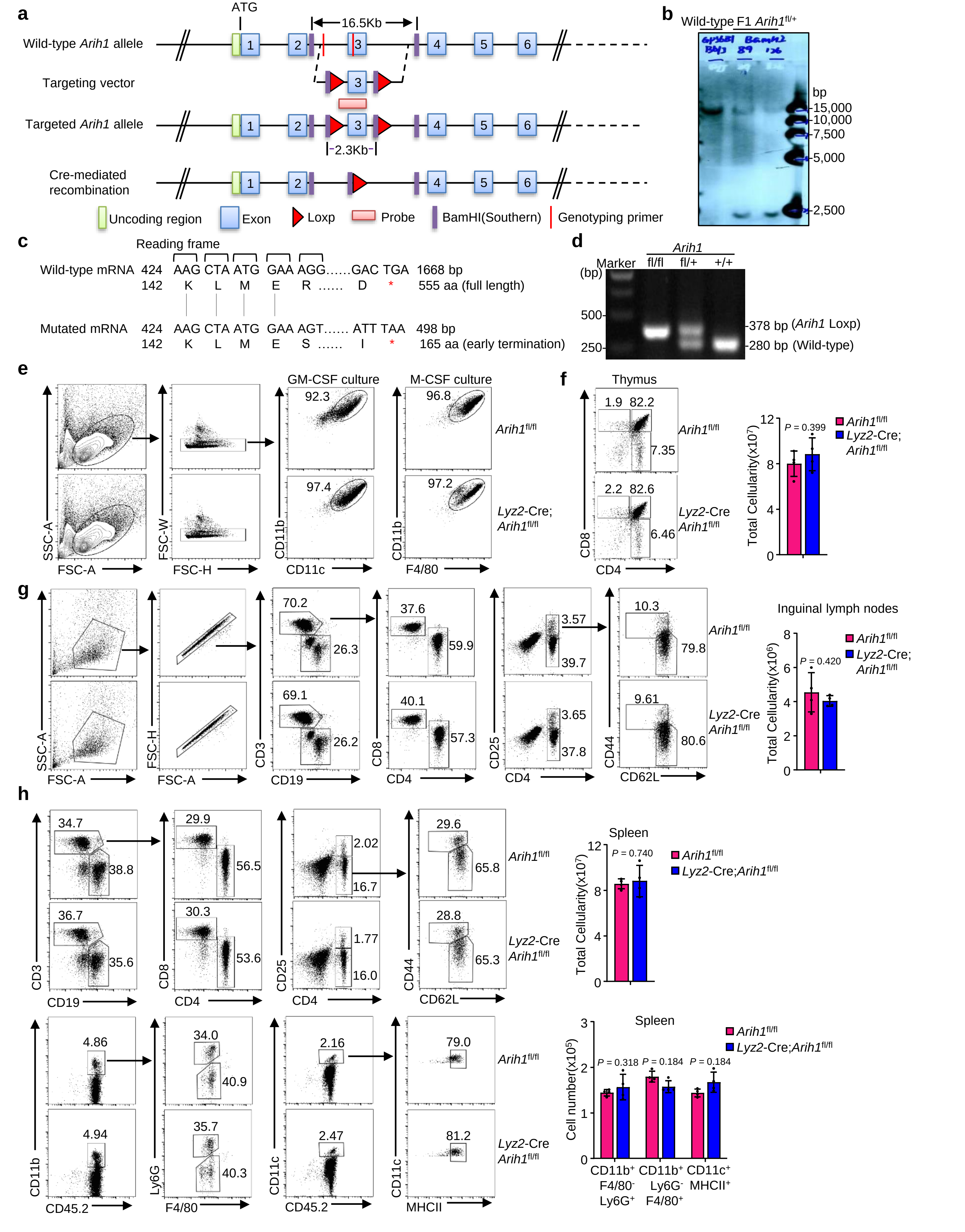

**Supplementary Figure 2 Generation of *Arih1*$^{fl/+}$ mice.**
(**a**) A scheme of CRIPSR/Cas9-mediated genome editing of the *Arih1* gene locus.
(**b**) Southern blot analysis of the generated F1 *Arih*$^{fl/+}$ mice.
(**c**) mRNA sequence and reading frame of wild-type and edited *Arih1* alleles.
(**d**) Genotyping analysis of the tail DNAs from *Arih*$^{fl/fl}$, *Arih*$^{fl/+}$ and unedited mice.
(**e**) Flow cytometry analysis of in vitro generated *Arih*$^{fl/fl}$ and *Lyz2*-Cre;*Arih*$^{fl/fl}$ BMDCs or BMDMs. The same Gating strategy was used in Fig. 2e and Supplementary Fig. 3f, 4e.
(**f-h**) Flow cytometry analysis of myeloid cells or lymphocytes and quantitative data in thymus (**f**), inguinal lymph nodes (**g**), and spleen (**h**) from *Arih*$^{fl/fl}$ and *Lyz2*-Cre;*Arih*$^{fl/fl}$ mice (n=4). Data are representative of two independent experiments (**e-h**). The same Gating strategy was used in Fig. 5e and Supplementary Fig. 2f, h.
Graphs show mean ± S.D. (n = 4 for **f-h**, biologically independent experiments). Statistical significance was determined using two-tailed Student's *t*-test in **f-h**. Source data are provided as a Source Data file.

## Supplementary Figure 3

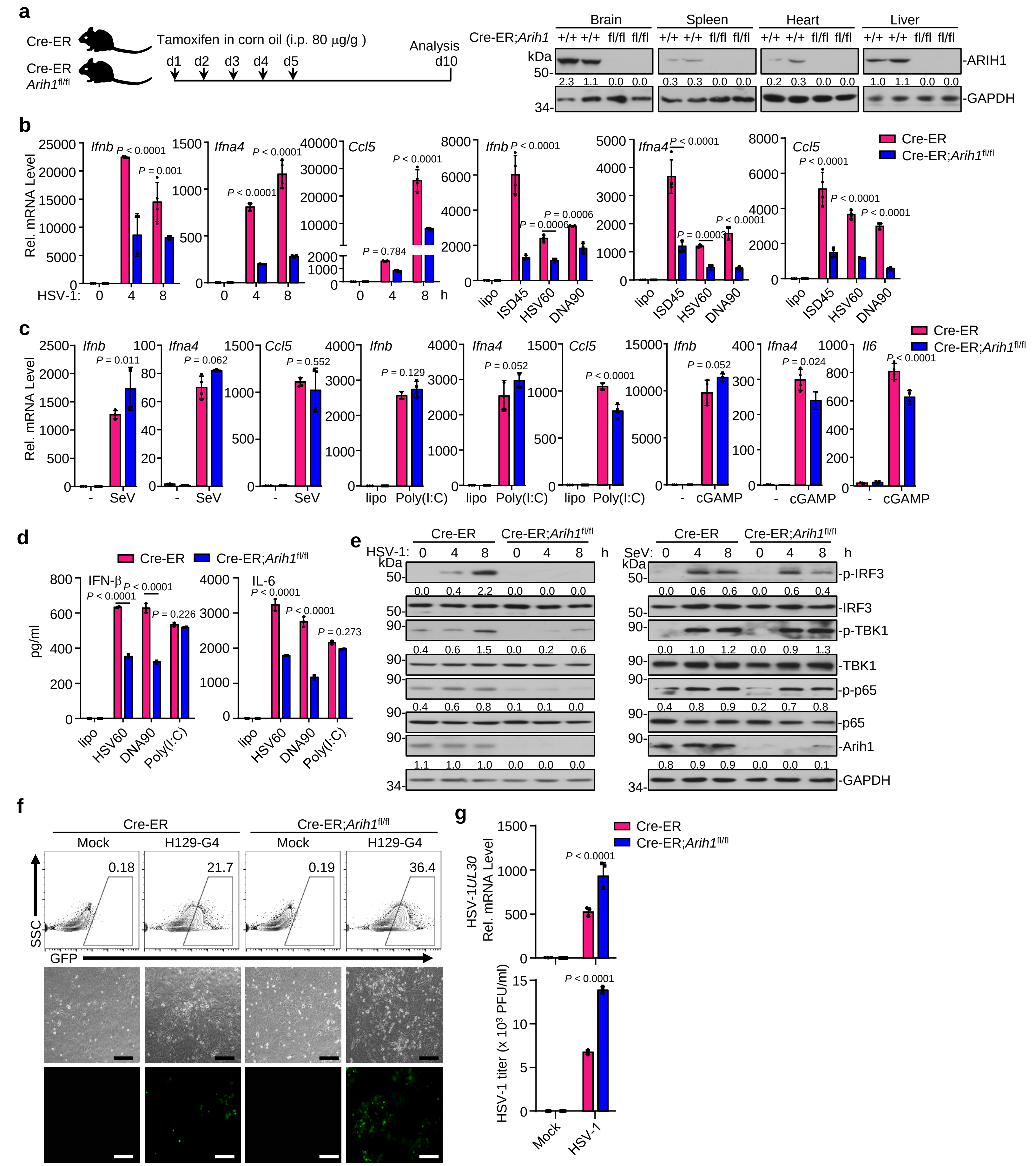

**Supplementary Figure 3 Knockout of ARIH1 inhibits cellular antiviral responses against HSV-1.**
(**a**) Immunblot analysis of ARIH1 and GAPDH in brains, spleens, hearts, and livers from Cre-ER and Cre-ER;*Arih*$^{fl/fl}$ mice that were intraperitoneally injected with tamoxifen (80 mg/kg in corn oil) for 5 successive days and rested for another 5 days.
(**b**) qRT-PCR analysis of *Ifnb*, *Ifna4* and *Ccl5* mRNA in Cre-ER and Cre-ER;*Arih1*$^{fl/fl}$ MLFs treated with 4-hydroxytamoxifen (4OHT, 1 μM) for 3 days followed by infection with HSV-1 for 0-8 h.
(**c**) qRT-PCR analysis of *Ifnb*, *Ifna4* and *Ccl5* mRNA in Cre-ER and Cre-ER;*Arih1*$^{fl/fl}$ MLFs treated as in (**a**) followed by infection with SeV for 0-8 h or transfection with poly(I:C) (1 μg) or cGAMP (1 μg) for 0-4 h.
(**d**) ELISA analysis of IFN-β and IL-6 in the supernatants of Cre-ER and Cre-ER;*Arih1*$^{fl/fl}$ MLFs treated as in (**a**) followed by transfection with HSV60, DNA90 and poly(I:C) (1 μg) for 12 h.
(**e**) Immunoblot analysis of total and phosphorylated (p-) IRF3,TBK1 and p65, Arih1 and GAPDH in Cre-ER and Cre-ER;*Arih1*$^{fl/fl}$ MLFs treated as in (**b**) followed by infection with HSV-1 (left panel) or SeV (right panel) for 0-8 h.
(**f**) Flow cytometric analysis (upper flow charts) and fluorescent microscopy imaging (lower images) of the replication of H129-G4 (MOI = 0.5) in Cre-ER and Cre-ER;*Arih1*$^{fl/fl}$ MLFs treated as in (**b**) that were left uninfected or infected with H129-G4 for 1 h followed by twice PBS wash and culture in full medium for 12 h. Scale bars represent 200 μm.
(**g**) qRT-PCR analysis of HSV-1 *UL30* mRNA (upper graph) and plaque assays (lower graph) analyzing HSV-1 titers in Cre-ER and Cre-ER;*Arih1*$^{fl/fl}$ MLFs treated as in (**b**) ) that were infected with HSV-1 (MOI = 0.5) for 1 h followed by twice PBS wash and culture in full medium for 12 h.
Data are representative of three (**b**, **c**) or two (**d-g**) independent experiments. Graphs show mean ± S.D. (n = 4 for **b-c**, **g**, or n = 3 for **d**, biologically independent experiments). Statistical significance was determined using two-tailed Student's *t*-test in **b-d**, **g**. The quantitative anaylsis is derive from the same experiment and that blots were processed in parallel in **e**. Source data are provided as a Source Data file.

## Supplementary Figure 4

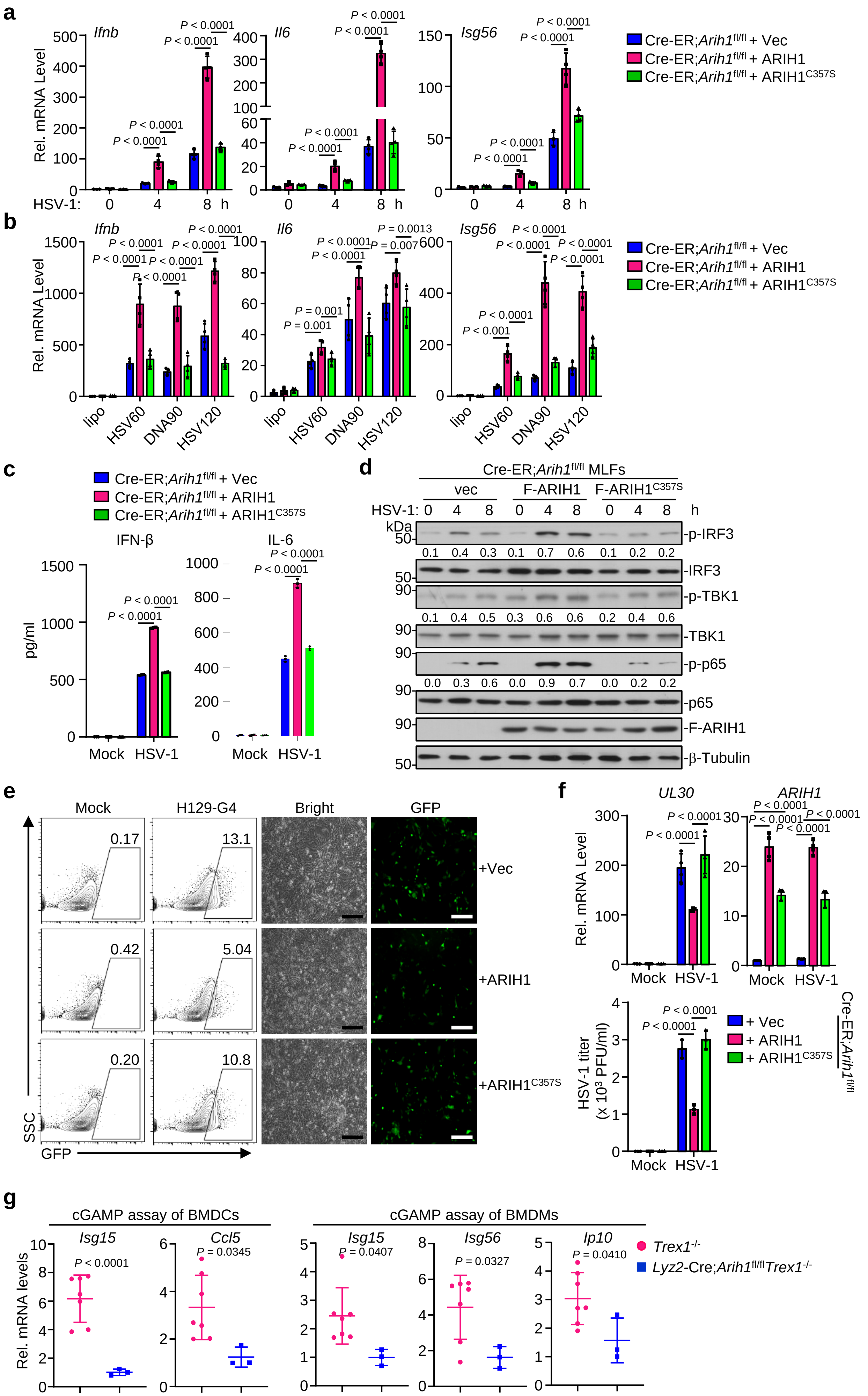

**Supplementary Figure 4 The enzyme activity of ARIH1 is required for antiviral signaling.**
(**a-c**) qRT-PCR analysis of *Ifnb*, *Il6* and *Isg56* mRNA (**a**, **b**) or ELISA analysis of IFN-β and IL-6 (**c**) in Cre-ER;*Arih1*$^{fl/fl}$ MLFs treated with 4-hydroxytamoxifen (4OH Tam, 1 μM) and reconstituted with empty vector (Vec), FLAG-ARIH1 or ARIH1$^{C357S}$ followed by infection with HSV-1 for 0-8 h (**a**) or 12 h (**c**) or transfection with HSV60, DNA90 or HSV120 (1 μg) for 8 h (**b**).
(**d**) Immunoblot analysis of total and phosphorylated (p-) IRF3,TBK1 and p65, FLAG-ARIH1 and β-tubulin in Cre-ER and Cre-ER;*Arih1*$^{fl/fl}$ MLFs treated as in (**a**) followed by infection with HSV-1 for 0-8 h.
(**e**) Flow cytometric analysis (left flow charts) and fluorescent microscopy imaging (right images) of the replication of H129-G4 (MOI = 0.5) in Cre-ER and Cre-ER;*Arih1*$^{fl/fl}$ MLFs treated as in (**a**) that were infected with H129-G4 for 1 h followed by twice PBS wash and culture in full medium for 12 h. Scale bars represent 200 μm.
(**f**) qRT-PCR analysis of HSV-1 *UL30* and *ARIH1* mRNA (upper graphs) and plaque assays (bottom graph) analyzing HSV-1 titers in Cre-ER and Cre-ER;*Arih1*$^{fl/fl}$ MLFs treated as in (**a**) that were infected with HSV-1 (MOI = 0.5) for 1 h followed by twice PBS wash and culture in full medium for 12 h.
(**g**) qRT-PCR analysis of *Isg15*, *Isg56* and *Ip10* in L929 cells to analyze the cGAMP activity in the homogenates of *Trex1*$^{-/-}$ and *Lyz2*-Cre;*Arih1*$^{fl/fl}$*Trex1*$^{-/-}$ BMDCs (left graphs) and BMDMs (right graphs) that were transfected with DNA120 (1 μg) for 6 h.
Data are representative of two independent experiments (**a-g**). Graphs show mean ± S.D. (n = 4 for **a-b**, **f** or n = 3 for **c**, or n = 7 and 3 for **g**, biologically independent experiments). Statistical significance was determined using two-tailed Student's *t*-test in **g**, or one-way ANOVA in **a-c**, **f**. The quantitative anaylsis is derive from the same experiment and that blots were processed in parallel in **d**. Source data are provided as a Source Data file.

# Supplementary Figure 5

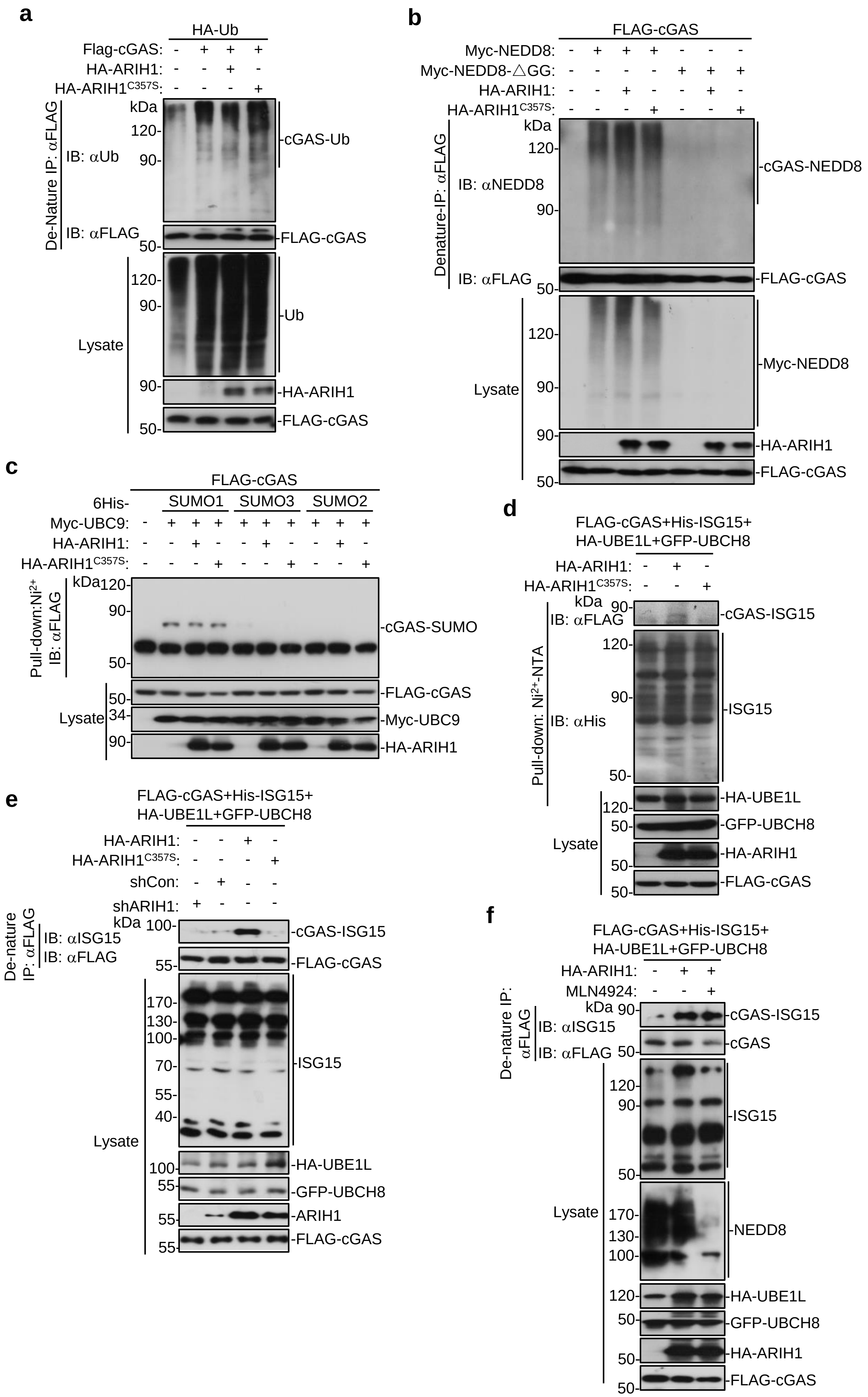

**Supplementary Figure 5 ARIH1 catalyzes mono-ISGylation but not other ubiquitin-like modifications of cGAS.**
(**a**) Denature-IP (with anti-FLAG) and immunoblot analysis (with anti-FLAG, anti-HA or anti-Ub) of HEK293 cells transfected with plasmids encoding an empty vector, HA-ARIH1 or ARIH1$^{C357S}$ together with FLAG-cGAS and HA-Ub for 24 h.
(**b**) Denature-IP (with anti-FLAG) and immunoblot analysis (with anti-FLAG, anti-HA, or anti-NEDD8) of HEK293 cells transfected with plasmids encoding an empty vector, HA-ARIH1 or ARIH1$^{C357S}$ together with FLAG-cGAS and Myc-NEDD8 or Myc-NEDD8△GG for 24 h.
(**c**) $Ni^{2+}$-NTA pull-down and immunoblot analysis (with anti-FLAG, anti-HA, or anti-Myc) of HEK293 cells transfected with plasmids encoding an empty vector, HA-ARIH1 or ARIH1$^{C357S}$ together with FLAG-cGAS, Myc-UBC9 and His-SUMO1, His-SUMO2 or His-SUMO3 for 24 h.
(**d**) $Ni^{2+}$-NTA pull-down and immunoblot analysis (with anti-FLAG, anti-HA, anti-GFP, or anti-His) of HEK293 cells transfected with plasmids encoding an empty vector, HA-ARIH1 or ARIH1$^{C357S}$ together with FLAG-cGAS, His-ISG15, GFP-UBCH8 and HA-UBE1L for 24 h.
(**e**) Denature-IP (with anti-FLAG) and immunoblot analysis (with anti-FLAG, anti-HA, anti-GFP or anti-ISG15) of HEK293 cells transfected with plasmids encoding an empty vector, HA-ARIH1, ARIH1$^{C357S}$, shCon, or shARIH1#2 together with FLAG-cGAS, His-ISG15, GFP-UBCH8 and HA-UBE1L for 24 h.
(**f**) Denature-IP (with anti-FLAG) and immunoblot analysis (with anti-FLAG, anti-HA, anti-GFP or anti-ISG15) of HEK293 cells transfected with plasmids encoding an empty vector, HA-ARIH1 or ARIH1$^{C357S}$ together with FLAG-cGAS, His-ISG15, GFP-UBCH8 and HA-UBE1L for 8 h followed by treatment with MLN4924 for 24 h.
Data are representative of three independent experiments (**a-f**). Source data are provided as a Source Data file.

# Supplementary Figure 6

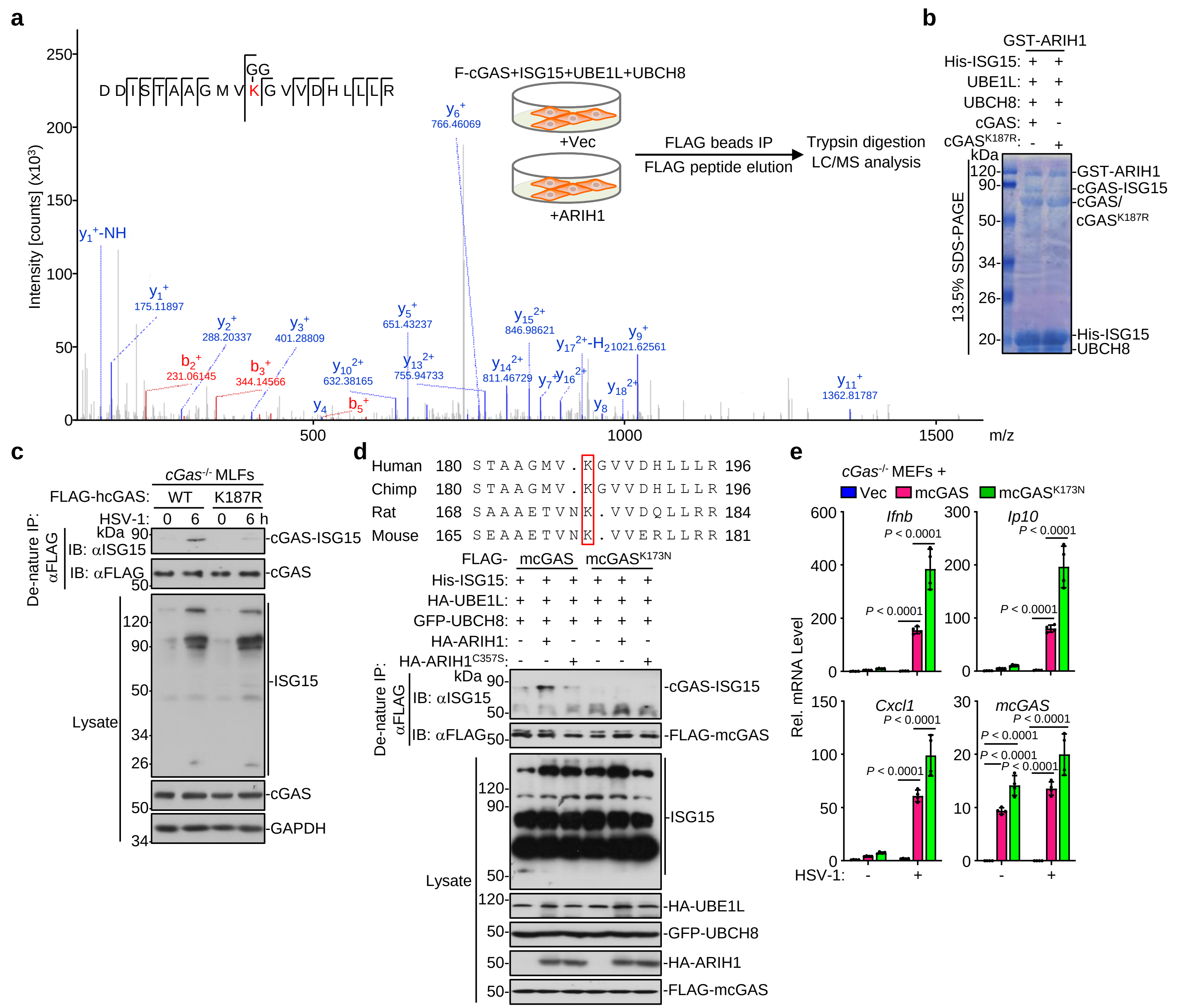


**Supplementary Figure 6 ARIH1 catalyzes mono-ISGylation of cGAS.**

(**a**) Strategy of identifying the ISGylated sites of cGAS. FLAG-cGAS was purified from HEK293 cells transfected with FLAG-cGAS, His-ISG15, HA-UBE1L and GFP-UBCH8 together with a empty control vector or HA-ARIH1 and followed by immunoprecipitation (with anti-FLAG agarose) and elution (with 3 × FLAG peptide). The eluted proteins was digested by trypsin and subsequently analyzed by LC/MS.

(**b**) In vitro ISGylation assay of cGAS or cGAS$^{K187R}$. Purified cGAS or cGAS$^{K187R}$ (5 µg) were incubated with GST-ARIH1 (5 µg), UBE1L (0.2 µg), UBCH8 (0.5 µg) and His-ISG15 (5 µg) in the presence of ATP (5 mM) at 37ºC for 2 h and subject to 13.5% SDS-PAGE followed by Coomassie brilliant blue staining.

(**c**) Denature-IP (with anti-FLAG) and immunoblot analysis (with anti-FLAG, anti-GAPDH or anti-ISG15) of *cGas*$^{-/-}$ MLFs reconstituted with FLAG-cGAS or cGAS$^{K187R}$ followed by HSV-1 infection for 0-6 h.

(**d**) An alignment of cGAS of different species (upper image). Denature-IP (with anti-FLAG) and immunoblot analysis (with anti-FLAG, anti-HA, anti-GFP or anti-ISG15) of HEK293 cells transfected with plasmids encoding an empty vector, HA-ARIH1 or ARIH1$^{C357S}$ together with His-ISG15, GFP-UBCH8 HA-UBE1L and FLAG-mcGAS or FLAG-mcGAS$^{K173N}$ for 24 h (lower blots).

(**e**) qPCR analysis of *Ifnb*, *Ip10*, *Cxcl1*, and *mcGAS* mRNA in *cGas*$^{-/-}$ MEFs reconstituted with an empty vector (Vec), mcGAS or mcGAS$^{K173N}$ followed by HSV-1 infection for 0-8 h.

Data are representative of three independent experiments (**b**-**e**). Graphs show mean ± S.D. (n = 4 for **e**, biologically independent experiments). Statistical significance was determined using one-way ANOVA in **e**. Source data are provided as a Source Data file.

# Supplementary Figure 7

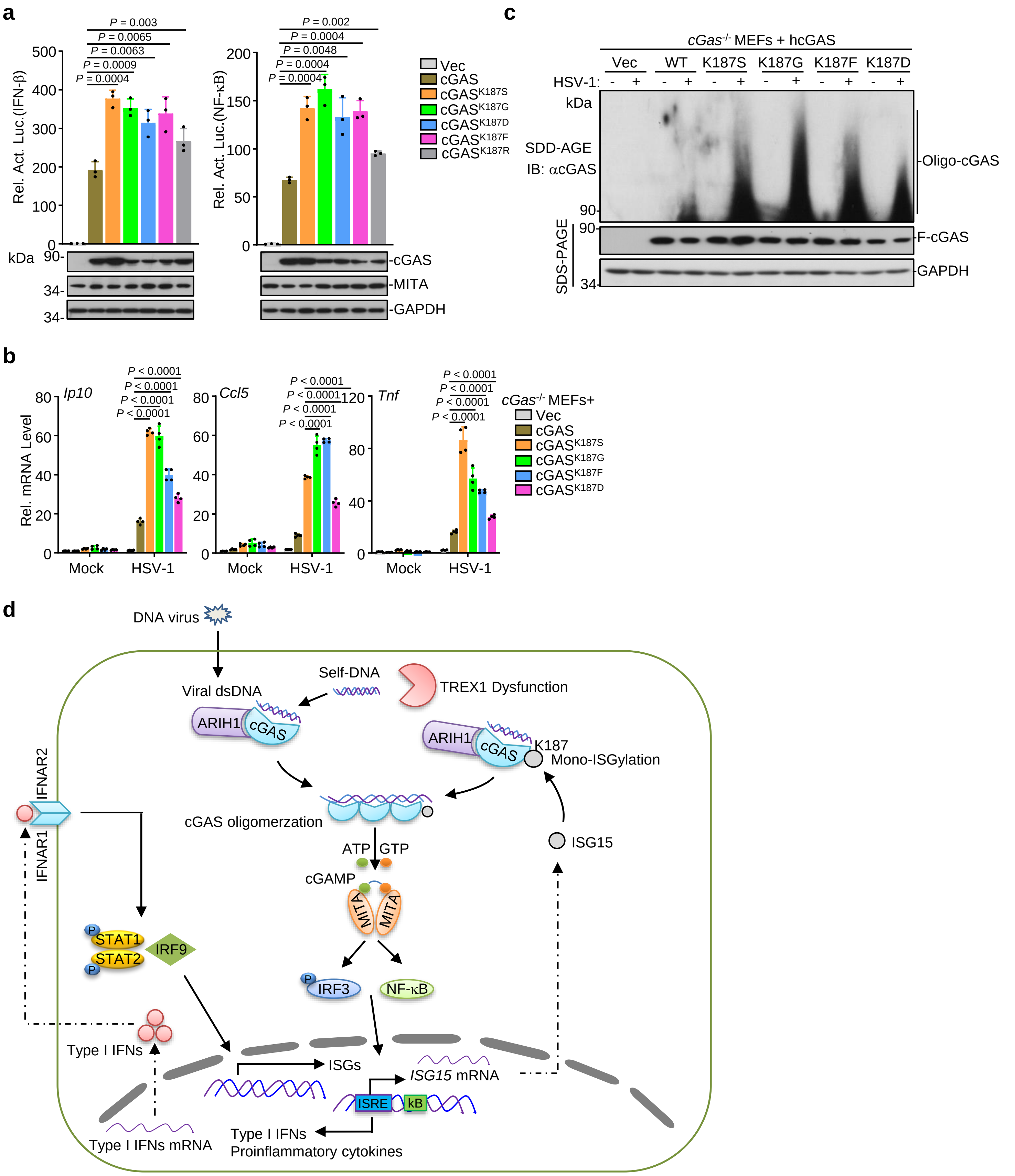


**Supplementary Figure 7 Lys 187 of cGAS inhibits its activation and oligomerization.**

(**a**) Luciferase reporter assays analyzing IFN-β and NF-κB promoter activity in HEK293 cells transfected with an empty vector (Vec), cGAS, cGAS$^{K187S}$, cGAS$^{K187G}$, cGAS$^{K187D}$, cGAS$^{K187F}$, or cGAS$^{K187R}$ together with FLAG-MITA for 24 hours.

(**b**) qRT-PCR analysis of *Ip10*, *Ccl5*, and *Tnf* in *cGas*$^{-/-}$ MEFs reconstituted with vec, cGAS, cGAS$^{K187S}$, cGAS$^{K187G}$, cGAS$^{K187D}$, or cGAS$^{K187F}$ followed by treatment with HSV-1 for 8 h.

(**c**) Immunoblot assays with SDD-AGE to analyze the aggregation of cGAS and with SDS-PAGE to analyze the expression levels of the indicated proteins in cells treated as in (**b**).

(**d**) A model on ARIH1-mediated mono-ISGylation of cGAS and regulation of cGAS-dependent antiviral immunity and autoimmunity.

Data are representative of three independent experiments (**a**-**c**). Graphs show mean ± S.D. (n = 3 for **a**, or n = 4 for **b**, biologically independent experiments). Statistical significance was determined using one-way ANOVA in **a**,**e**. Source data are provided as a Source Data file.

Supplementary Table 1. antibody information

| Antibody | Source & Identifier | Dilution |
|---|---|---|
| mouse control IgG | Santa Cruz Biotechnology, sc-2025 | 1/1000 |
| rabbit control IgG | Millipore, 12-370 | 1/1000 |
| HRP-conjugated goat-anti mouse or rabbit IgG | Thermo Scientific, PA1-86717 and SA1-9510 | 1/1000 |
| mouse anti-FLAG | Sungene, KM8002 | 1/5000 |
| anti-GFP | Sungene, KM8009 | 1/5000 |
| anti-β-Actin | Sungene, KM9001 | 1/10000 |
| anti-GAPDH | Sungene, KM9002 | 1/10000 |
| anti-Tubulin | Sungene, KM9003 | 1/10000 |
| anti-Myc | Sungene, KM8003 | 1/10000 |
| anti-HA | Covance, MMS-101R | 1/10000 |
| anti-Ubiquitin | Santa Cruz Biotechnology, sc-8017 | 1/1000 |
| anti-TBK1 | Abcam, 96328-11 | 1/5000 |
| anti-p-TBK1 | Abcam, 109272 | 1/1000 |
| anti-IRF3 | Santa Cruz Biotechnology, sc-33641 | 1/5000 |
| anti-p-IRF3 | Cell Singling Technologies, 4947S | 1/1000 |
| anti-p65 | Santa Cruz Biotechnology, sc-8008 | 1/2000 |
| anti-p-p65 | Cell Singling Technologies, 3033S | 1/1000 |
| anti-hcGAS | Cell Singling Technologies, 83623 | 1/1000 |
| anti-mcGAS | Cell Singling Technologies, 31659 | 1/1000 |
| anti-ARIH1 | Abcam, ab3891 | 1/1000 |
| anti-ISG15 | Abclonal, A2416 | 1/1000 |
| anti-NEDD8 | Abclonal, A1163 | 1/1000 |
| anti-CD3 | Biolegend, 100312 | 1/300 |
| anti-CD19 | Biolegend, 152404 | 1/300 |
| anti-GL7 | Biolegend, 144610 | 1/300 |
| anti-FAS | Biolegend, 152608 | 1/300 |
| anti-CD4 | Biolegend, 558107 | 1/300 |
| anti-CD8 | Biolegend, 100734 | 1/300 |
| anti-CD44 | Biolegend, 103012 | 1/300 |
| anti-CD62L | Sungene, M10621-02E | 1/300 |
| anti-CD25 | Sungene, M10251-09B | 1/300 |
| anti-CD11b | Sungene, M10117-02E | 1/300 |
| anti-CD45.2 | Biolegend, 109806 | 1/300 |
| anti-F4/80 | Biolegend, 123110 | 1/300 |
| anti-Ly6G | biolegend, 127624 | 1/300 |
| anti-CD11c | Biolegend, 117328 | 1/300 |
| anti-MHCII | Sungene, M100M2-11C | 1/300 |
| anti-CD16/32 | Sungene, M10161-02B | 1/300 |

## Supplementary Table 2. sequences of DNA ligands

| DNA ligands | Forward | Reverse |
|---|---|---|
| ISD45 | TACAGATCTACTAGTGATCTATGACTGAT | TGTAGATCATGTACAGATCAGTCATAGAT |
| | CTGTACATGATCTACA | CACTAGTAGATCTGTA |
| HSV60 | TAAGACACGATGCGATAAAATCTGTTTGT | GCTAGGGCAATTTGTACCCTTAATAAATT |
| | AAAATTTATTAAGGGTACAAATTGCCCTAGC | TTACAAACAGATTTTATCGCATCGTGTCTTA |
| DNA90 | TACAGATCTACTAGTGATCTATGACTGATC | TGTAGATCATGTACAGATCAGTCATAGATC |
| | TGTACATGATCTACATACAGATCTACTAGT | ACTAGTAGATCTGTATGTAGATCATGTACA |
| | GATCTATGACTGATCTGTACATGATCTACA | GATCAGTCATAGATCACTAGTAGATCTGTA |
| HSV120 | AGACGGTATATTTTTGCGTTATCACTGTCCC | AAGTCCTCCAAAAAACCCGCCACAAATAAA |
| | GGATTGGACACGGTCTTGTGGGATAGGCAT | AAGGGGTTAACCCAATATGCCTTCTGGGCAT |
| | GCCCAGAAGGCATATTGGGTTAACCCCTTT | GCCTATCCCACAAGACCGTGTCCAATCCGGG |
| | TTATTTGTGGCGGGTTTTTTGGAGGACTT | ACAGTGATAACGCAAAAATATACCGTCT |

Supplementary Table 3. sequences of qPCR primers

| Gene | Forward | Reverse |
|---|---|---|
| *β-Actin* | CACCATTGGCAATGAGCGGTTC | AGGTCTTTGCGGATGTCCACGT |
| *cGAS* | AGAAGGCCTGCGCATTCAAA | GCCGCCATGTTTCTTCTTGGA |
| *CXCL1* | AGCTTGCCTCAATCCTGCATCC | TCCTTCAGGAACAGCCACCAGT |
| *IFN-β* | TTGTTGAGAACCTCCTGGCT | TGACTATGGTCCAGGCACAG |
| *IL6* | AGACAGCCACTCACCTCTTCAG | TTCTGCCAGTGCCTCTTTGCTG |
| *IP10* | GGTGAGAAGAGATGTCTGAATCC | GTCCATCCTTGGAAGCACTGCA |
| *ISG15* | AGGACAGGGTCCCCCTTGCC | CCTCCAGCCCGCTCACTTGC |
| *ISG56* | TCATCAGGTCAAGGATAGTC | CCACACTGTATTTGGTGTCTAGG |
| *ARIH1* | ACTTTGATGGAAACCTGGAGAA | GCCAGTGAAATACGAGTTAGGG |
| *CCL5* | GGCAGCCCTCGCTGTCATCC | GCAGCAGGGTGTGGTGTCCG |
| *TNF* | GCCGCATCGCCGTCTCCTAC | CCTCAGCCCCCTCTGGGGTC |
| *UL30* | CATCACCGACCCGGAGAGGGAC | GGGCCAGGCGCTTGTTGGTGTA |
| m*Isg56* | GAAGTCCTTTGCTTGGAGGA | AGCCCAAGAAGGCTGGTACT |
| m*Ifnb* | TACAACAGATACGCCTGGAT | AGTCCGCCTCTGATGCTTAA |
| m*Il6* | ACAAAGCCAGAGTCCTTCAGA | TCCTTAGCCACTCCTTCTGT |
| m*Ccl5* | ATATGGCTCGGACACCACTC | CCACTTCTTCTCTGGGTTGG |
| m*cGAS* | TGTCAGTGTGGAGAAGGAAAAA | CAGCCAGCCTTGAATAGGTAG |
| m*Arih1* | GGTGAAACAGAAGATGGAGGAG | TTTGAGGTAGAAAGCGAAGACA |
| m*Ifna4* | AGGATCACTGTGTACCTGAGA | TCTCCACACTTTGTCTCAGGA |
| m*Ip10* | GTGAGAATGAGGGCCATAGG | TTTTTGGCTAAACGCTTTCAT |
| m*Isg15* | GGCCACAGCAACATCTATGA | ACTGGGGCTTTAGGCCATAC |
| m*Cxcl1* | CACTCAAGAATGGTCGCGAG | GTTGTCAGAAGCCAGCGTTC |
| m*β-actin* | ACGGCCAGGTCATCACTATT | TGGCATAGAGGTCTTTACGGA |
