# Supplementary material for: The E3 ubiquitin ligase ARIH1 promotes antiviral immunity and autoimmunity by inducing mono-ISGylation and oligomerization of cGAS
Source: Nat Commun. 2022 Oct 10;13:5973. doi: 10.1038/s41467-022-33671-5 (PMC9551088; doi:10.1038/s41467-022-33671-5)
Supplement: Supplementary file 2 — Reporting Summary [file 41467_2022_33671_MOESM2_ESM.pdf]

nature portfolio

Corresponding author(s): Bo Zhong, Zhidong Zhang, Dandan Lin.

Last updated by author(s): Sep 23, 2022

Reporting Summary

Nature Portfolio wishes to improve the reproducibility of the work that we publish. This form provides structure for consistency and transparency in reporting. For further information on Nature Portfolio policies, see our Editorial Policies and the Editorial Policy Checklist.

Statistics

For all statistical analyses, confirm that the following items are present in the figure legend, table legend, main text, or Methods section.

n/a	Confirmed	
☐	☒	The exact sample size (*n*) for each experimental group/condition, given as a discrete number and unit of measurement
☐	☒	A statement on whether measurements were taken from distinct samples or whether the same sample was measured repeatedly
☐	☒	The statistical test(s) used AND whether they are one- or two-sided<br>*Only common tests should be described solely by name; describe more complex techniques in the Methods section.*
☒	☐	A description of all covariates tested
☒	☐	A description of any assumptions or corrections, such as tests of normality and adjustment for multiple comparisons
☐	☒	A full description of the statistical parameters including central tendency (e.g. means) or other basic estimates (e.g. regression coefficient) AND variation (e.g. standard deviation) or associated estimates of uncertainty (e.g. confidence intervals)
☐	☒	For null hypothesis testing, the test statistic (e.g. *F*, *t*, *r*) with confidence intervals, effect sizes, degrees of freedom and *P* value noted<br>*Give P values as exact values whenever suitable.*
☒	☐	For Bayesian analysis, information on the choice of priors and Markov chain Monte Carlo settings
☒	☐	For hierarchical and complex designs, identification of the appropriate level for tests and full reporting of outcomes
☒	☐	Estimates of effect sizes (e.g. Cohen's *d*, Pearson's *r*), indicating how they were calculated

Our web collection on statistics for biologists contains articles on many of the points above.

Software and code

Policy information about availability of computer code

Data collection	BD FACSDiVa Software v8.0.1.1 for FACS;Bio-Rad CFX Manager 3.1 for qRT-PCR.
Data analysis	GraphPad Prism 9.0.0 for graphs and statistical analysis; FlowJo X 10.0.7r2 for FACS plots; Skyline 21.1.0.278 for LC-MS analysis.

For manuscripts utilizing custom algorithms or software that are central to the research but not yet described in published literature, software must be made available to editors and reviewers. We strongly encourage code deposition in a community repository (e.g. GitHub). See the Nature Portfolio guidelines for submitting code & software for further information.

Data

Policy information about availability of data

All manuscripts must include a data availability statement. This statement should provide the following information, where applicable:

- Accession codes, unique identifiers, or web links for publicly available datasets
- A description of any restrictions on data availability
- For clinical datasets or third party data, please ensure that the statement adheres to our policy

All data are included in the Supplemental Information or available from the authors upon reasonable requests, as are unique reagents used in this Article. The raw numbers for charts and graphs including Figs. 1b-f, 2a-d, 2f, 3a-e, 3g-k, 4b-e, 5, 6 and 7 and Supplementary Figs. 1, 2f-h, 3a-e, 3g, 4a-d, 4f-g, 5, 6b-e, 7a-c. are available in the Source Data file whenever possible. Source data are provided with this paper.

Human research participants

Policy information about studies involving human research participants and Sex and Gender in Research.

Reporting on sex and gender	N/A
Population characteristics	N/A
Recruitment	N/A
Ethics oversight	N/A

Note that full information on the approval of the study protocol must also be provided in the manuscript.

Field-specific reporting

Please select the one below that is the best fit for your research. If you are not sure, read the appropriate sections before making your selection.

- [x] Life sciences
- [ ] Behavioural & social sciences
- [ ] Ecological, evolutionary & environmental sciences

For a reference copy of the document with all sections, see nature.com/documents/nr-reporting-summary-flat.pdf

Life sciences study design

All studies must disclose on these points even when the disclosure is negative.

Sample size	No statistical methods were used to determine sample size needed. For in vivo animal experiments, sample size was determined on the basis of prior knowledge of variability of similar experiments in our laboratory and chosen as large as possible while taking into account the experimental effort required to generate the respective data. For in vitro experiments, a minimum of two samples were analyzed, which were biologically repeated for at least two times.
Data exclusions	No data was excluded from our study.
Replication	The experiments were performed with 2-3 independent replications. The replication numbers were described in the corresponding figure legends. For each replication, age- and sex-matched mice were used.
Randomization	For in vitro experiments, cells were randomly allocated into control and experimental groups. For in vivo experiments, age and sex-matched mice were randomized into all experimental groups.
Blinding	For in vivo infection experiments the researchers were not blinded since they need to treat the mice first and then perform the infection experiments afterwards. However, at least two to three researchers performed the experiments and collected the samples at the same time to exclude the bias. For experiments other than mouse infection, the researchers were not blinded during data collection and analysis. However, the experiments are repeated by independent researchers to validate the results. Cellular and biochemical experiments were not performed in a blinding manner.

Reporting for specific materials, systems and methods

We require information from authors about some types of materials, experimental systems and methods used in many studies. Here, indicate whether each material, system or method listed is relevant to your study. If you are not sure if a list item applies to your research, read the appropriate section before selecting a response.

Materials & experimental systems

n/a	Involved in the study	
[ ]	[x]	Antibodies
[ ]	[x]	Eukaryotic cell lines
[x]	[ ]	Palaeontology and archaeology
[ ]	[x]	Animals and other organisms
[x]	[ ]	Clinical data
[x]	[ ]	Dual use research of concern

Methods

n/a	Involved in the study	
[x]	[ ]	ChIP-seq
[ ]	[x]	Flow cytometry
[x]	[ ]	MRI-based neuroimaging

Antibodies

Antibodies used	mouse control IgG (Santa Cruz Biotechnology, sc-2025, 1/1000), rabbit control IgG (Millipore, 12–370, 1/1000), HRP-conjugated goat-anti mouse or rabbit IgG (Thermo Scientific, PA1-86717 and SA1-9510, 1/1000), mouse anti-FLAG (Sungene, KM8002, 1/5000), anti-GFP (Sungene, KM8009, 1/5000), anti-b-Actin (Sungene, KM9001, 1/10000), anti-GAPDH (Sungene, KM9002, 1/10000), anti-Tubulin (Sungene, KM9003, 1/10000), anti-Myc (Sungene, KM8003, 1/10000), anti-HA (COVANCE, MMS-101R, 1/10000), anti-Ubiquitin (Santa Cruz Biotechnology, sc-8017, 1/1000); anti-TBK1 (Abcam, 96328-11, 1/5000), anti-p-TBK1 (Abcam, 109272, 1/1000), anti-IRF3 (Santa Cruz Biotechnology, sc-33641, 1/5000), anti-p-IRF3 (Cell Singling Technologies, 4947S, 1/1000), anti-p65 (Santa Cruz Biotechnology, sc-8008, 1/2000), anti-p-p65 (Cell Singling Technologies, 3033S, 1/1000), anti-hcGAS (Cell Singling Technologies, 83623, 1/1000), anti-mcGAS (Cell Singling Technologies, 31659, 1/1000), anti-ARIH1 (Abcam, ab3891, 1/1000), anti-ISG15 (Abclonal, A2416, 1/1000), anti-NEDD8 (Abclonal, A1163, 1/1000), anti-CD3 (Biolegend, 100312, 1/300), anti-CD19 (Biolegend, 152404, 1/300), anti-GL7 (Biolegend, 144610, 1/300), anti-FAS (Biolegend, 152608, 1/300), anti-CD4 (Biolegend, 558107, 1/300), anti-CD8 (Biolegend, 100734, 1/300), anti-CD44 (Biolegend, 103012, 1/300), anti-CD62L (Sungene, M10621-02E, 1/300), anti-CD25 (Sungene, M10251-09B, 1/300), anti-CD11b (Sungene, M10117-02E, 1/300), anti-CD45.2(Biolegend, 109806, 1/300), anti-F4/80 (Biolegend, 123110, 1/300), anti-Ly6G (Biolegend, 127624, 1/300), anti-CD11c (Biolegend, 117328, 1/300), anti-MHCII (Sungene, M100M2-11C, 1/300), anti-CD16/32 (Sungene, M10161-02B, 1/300).
Validation	anti-ARIH1, ab3891,WB, https://www.abcam.cn/arih1-antibody-ab3891.html; anti-ISG15, A2416, https://abclonal.com.cn/catalog/A2416; anti-NEDD8, A1163, https://abclonal.com.cn/catalog/A1163; Other antibodies were used and validated in our previously publications(Sun H et al.,2017,Nat Commun 2017, 8, 15534; Liuyu T, et al., Cell Res, 2019, 29(1):67-79; Zhang Q et al., Cell Res, 2020,30(10):914-927.)

Eukaryotic cell lines

Policy information about cell lines and Sex and Gender in Research

Cell line source(s)	THP-1, HEK293, L929, HFF and Vero cells were from the American Type Culture Collection (Zhang Q et al., Cell Res, 2020, 30 (10): 914- 927.).THP-1: human acute monocytic leukemia cells both male and female. HEK293: human embryonic kidney cells both male and female. L929: mouse fibrolast cell line both male and female. HFF: male foreskin fibrolast. Vero: kidney epithelial cells extracted from an African green monkey. Mouse bone marrow-derived dendritic cells (BMDC), Mouse bone marrow-derived macrophages (BMDM) and mouse lung fibroblasts (MLF) were generated as described in the methods section. cGas-/- MEFs were provided by Drs. Ming-Ming Hu and Hong-Bing Shu (Wuhan University).
Authentication	These cells were authenticated by STR locus analysis.
Mycoplasma contamination	The cells were tested Mycoplsma negative.
Commonly misidentified lines (See ICLAC register)	No commonly misidentified cell lines were used in the study.

Animals and other research organisms

Policy information about studies involving animals; ARRIVE guidelines recommended for reporting animal research, and Sex and Gender in Research

Laboratory animals	The Arih1fl/+ mice were generated by GemPharmatech Co. Ltd through CRISPR/Cas9-mediated gene editing. In brief, guide RNAs (5'-GGCAGGAGCAGGCGAGCCCT-3' and 5'-AAGTAAGTGATATAGCCCCC-3') were obtained through in vitro transcription and purification. The gRNAs were incubated with purified Cas9 protein and injected into the fertilized eggs (at the one-cell stage) together with the targeting vector with two loxp sites flanking the exon 3 of the Arih1 gene. The injected fertilized eggs were cultured to the two-cell stage followed by transplantation into pseudopregnant mice. The targeted genomes of F0 mice were amplified by PCR and sequenced and the chimeras were crossed with wild-type C57BL/6 mice to obtain F1 Arih1fl/+ mice. Southern blot analysis was conducted with the tail DNA from F1 mice to confirm correct recombination and exclude random insertions of the targeting vector. Lyz2-Cre mice (B6/JNju-Lyz2em1Cin(iCre)/Nju, stock number: T003822) were purchased from the Nanjing Biomedical Research Institute of Nanjing University. Trex1+/- mice were described previously25. Cre-ER mice (B6.129-Gt(ROSA)26Sortm1(cre/ERT2)Tyj, stock number: 008463) were from the Jackson Laboratory and kindly provided by Dr. Chen Dong (Tsinghua University). Arih1fl/+ mice were crossed with Cre-ER, Lyz2-Cre, or Trex1+/- mice to obtain Cre-ER;Arih1fl/fl, Lyz2-Cre;Arih1fl/fl, and Lyz2-Cre;Arih1fl/flTrex1-/- mice, respectively. All genetic models were on the C57BL/6 background. Mice including both sexes, between the ages of 5-8 weeks were used for all described experiments. 5-week old, gender-matched Trex1-/- and Lyz2-Cre;Arih1fl/flTrex1-/- mice were used for experiments. 6 to 8-week old, gender-matched mice were used for all remaining experiments.
Wild animals	This study did not involve wild animals.
Reporting on sex	There was no sex bias in the animals used in this study.
Field-collected samples	This study did not involve samples collected from the field.
Ethics oversight	All animal experiments were in accordance with protocols approved by the Institutional Animal Care and Use Committee of Wuhan University.

Note that full information on the approval of the study protocol must also be provided in the manuscript.

Flow Cytometry

Plots

Confirm that:

- [x] The axis labels state the marker and fluorochrome used (e.g. CD4-FITC).
- [x] The axis scales are clearly visible. Include numbers along axes only for bottom left plot of group (a 'group' is an analysis of identical markers).
- [x] All plots are contour plots with outliers or pseudocolor plots.
- [x] A numerical value for number of cells or percentage (with statistics) is provided.

Methodology

Sample preparation	Single cell suspensions were incubated with fluorochrome-conȷ ugated antibodies against surface markers in PBS containing 1.5% FBS for 20 min at 4 C and then washed. Cells were then fixed for 30 min at 4 C using Biolegend Cytofix/Cytoperm and washed twice followed by flow cytometry analysis.The detailed experimental procedures were described in the methods session.
Instrument	BD Fortessa and Celesta
Software	Windows Flowjo 10.6.2 for data analysis; BD FACSDiVa v8.0.1.1 for cell collection.
Cell population abundance	All cell populations tested were abundant (at least 20,000 events collected).
Gating strategy	Forward versus side scatter (FSC vs SSC) gating was used to identify cells of interest and exclude debris and dead cells. Also, A forward scatter height (FSC-W/A) vs. forward scatter area (FSC-H) density plot was used to exclude doublets. For cytokine measurement, we used non-restimulated samples as negative controls.

- [x] Tick this box to confirm that a figure exemplifying the gating strategy is provided in the Supplementary Information.
